# Ultrasensitive photoelectric detection with room temperature extremum

**DOI:** 10.1038/s41377-024-01701-0

**Published:** 2025-02-26

**Authors:** Tuntan Wu, Yongzhen Li, Qiangguo Zhou, Qinxi Qiu, Yanqing Gao, Wei Zhou, Niangjuan Yao, Junhao Chu, Zhiming Huang

**Affiliations:** 1 State Key Laboratory of Infrared Physics, Shanghai Institute of Technical Physics, Chinese Academy of Sciences, Shanghai, 200083 China; 2 Hangzhou Institute for Advanced Study, University of Chinese Academy of Sciences, Hangzhou, 310024 China; 3 University of Chinese Academy of Sciences, Beijing, 100049 China; 4 Institute of Optoelectronics, Fudan University, Shanghai, 200438 China; 5 Key Laboratory of Space Active Optical-Electro Technology, Chinese Academy of Sciences, Shanghai, 200083 China

**Keywords:** Optoelectronic devices and components, Photonic devices

## Abstract

Room-temperature photodetection holds pivotal significance in diverse applications such as sensing, imaging, telecommunications, and environmental remote sensing due to its simplicity, versatility, and indispensability. Although different kinds of photon and thermal detectors have been realized, high sensitivity of photodetection with room temperature extremum is not reported until now. Herein, we find evident peaks in the photoelectric response originated from the anomalous excitonic insulator phase transition in tantalum nickel selenide (Ta_2_NiSe_5_) for room-temperature optimized photodetection from visible light to terahertz ranges. Extreme sensitivity of photoconductive detector with specific detectivity (D*) of 5.3 × 10^11^ cm·Hz^1/2^·W^−^^1^ and electrical bandwidth of 360 kHz is reached in the terahertz range, which is one to two orders of magnitude improvement compared to that of the state-of-the-art room-temperature terahertz detectors. The van der Waals heterostructure of Ta_2_NiSe_5_/WS_2_ is further constructed to suppress the dark current at room temperature with much improved ambient D* of 4.1 × 10^12^ cm·Hz^1/2^·W^−1^ in the visible wavelength, rivaling that of the typical photodetectors, and superior photoelectric performance in the terahertz range compared to the photoconductor device. Our results open a new avenue for optoelectronics via excitonic insulator phase transition in broad wavelength bands and pave the way for applications in sensitive environmental and remote sensing at room temperature.

## Introduction

Photodetection shows extremely important and extensive applications in diverse fields including imaging^1^, quantum information^2,3^, communication^4,5^, wearable electronics^6,7^ and space science^8^. The capacity to transform light into electronic signals holds crucial significance in photodetection, with the temperature range for this transformation spanning from near absolute zero Kelvin to above room temperature. Among them, the paramount significance of room temperature broadband photodetection lies in its ability to enable the compact systems for numerous optoelectronics technologies, its suitability for a wide range of applications, and its low-cost. However, high sensitivity of detection approach at room temperature remains considerably lacking from infrared to terahertz range. In addition, although mature development of room temperature detection has been achieved in the ultraviolet^9^, visible light^10^ and millimeter wave^11^, high sensitivity of photodetection optimized near room temperature is not reported until now.

One crucial approach is to operate photodetectors under the phase-transition temperature of the material along with obvious change of electrical and optical properties. Materials demonstrating phase transitions have consistently been a central focus in photodetector research endeavors^12–14^. Typically, in proximity to the transition temperature, those materials manifest distinctive electronic and photonic properties, thereby presenting myriad opportunity for the advancement of photoelectric detection. However, high sensitivity of photodetection with ambient extremum is not found. On one hand, the operational temperature of these detectors is constrained by the phase transition temperature (*T*_C_), and the optimal operating temperature for peak performance does not fall within the ambient temperature range. For instance, superconductor-insulator-superconductor mixer^15,16^ and superconducting transition edge detector (TES)^17^, based on superconductor-to-normal phase transitions, achieve ultra-high sensitivity detection of terahertz photons. Nonetheless, constrained by the *T*_C_ associated with superconducting phase transitions, these detectors typically operate at extremely low temperatures, rendering them impractical for operation under ambient conditions. The titanium diselenide (TiSe_2_) detector based on electromagnetic induced well (EIW) effect^18^, endowed with a charge density wave (CDW), attains heightened performance in terahertz light detection following the Peierls phase transition, attributable to the abrupt alteration in its transport properties. Nevertheless, its optimal performance materializes at temperatures below 200 K, signifying a substantial disparity from ambient conditions. On the other hand, certain materials exhibit *T*_C_ within the ambient range, for instance, vanadium oxide (VO_x_)^19–21^, featuring the Mott phase transition, undergoes a sudden increase in resistance from metal to insulator in the vicinity of room temperature. Similarly, barium strontium titanate^22^, possessing ferroelectric phase transition, manifests spontaneous polarization below its Curie temperature near room temperature, concomitant with a rapid decline in dielectric constant. Bolometers and pyroelectric detectors based on these materials can operate at room temperature, nevertheless, constrained by their thermal detection mechanisms, they typically exhibit relatively inferior response speed at ambient conditions with very low electrical bandwidth. Consequently, the identification of materials with *T*_C_ within the room temperature range, capable of attaining optimal photoelectric detection performance at room temperature, holds paramount significance for the advancement of room-temperature optoelectronics.

Tantalum nickel selenide (Ta_2_NiSe_5_) is a material undergoing a semimetal to excitonic insulator (EI) phase transition at 326 K^23–26^. EI is a unique state of matter that emerges from Bose-Einstein condensation (BEC) of electron-hole pairs in solids^27,28^. Within EI phase, a substantial number of excitons are created, and these excitons can condense into a macroscopic coherent state, thereby giving rise to the establishment of an insulating state with a finite energy bandgap^29,30^. Unlike conventional insulators where electrons are localized, EIs are characterized by the collective motion and behavior of excitons^31^, can exert effects on photoelectric phenomena in a collective fashion. Furthermore, Ta_2_NiSe_5_ serves as a layered material that can be readily obtained in nanosheets through mechanical exfoliation. This facilitates the exploration of two-dimensional scale photoelectric devices and can be employed to construct van der Waals (vdW) heterostructure without the lattice-mismatch problem.

In this article, we firstly achieve optimized room-temperature response peak harnessing the EI phase transition in Ta_2_NiSe_5_. High-performance, ambient photodetection is realized spanning from visible light (VIS), near infrared (NIR), short wavelength infrared (SWIR) to terahertz (THz), thereby demonstrating Ta_2_NiSe_5_ as an exceptionally promising material ideally suited for ultrasensitive room-temperature photodetection. We also construct a vdW heterojunction photodetector based on Ta_2_NiSe_5_ and WS_2_, effectively suppressing dark current while enhancing photoelectric response of Ta_2_NiSe_5_. In the terahertz range, the heterojunction photodetector achieves a specific detectivity D^*^ of 7.0 × 10^11^ cm·Hz^1/2^·W^−^^1^, which represents 1–2 orders improvement compared to that of the state-of-the-art room-temperature terahertz devices. Furthermore, the optimized D^*^ reaches an impressive value of 4.1 × 10^12^ cm·Hz^1/2^·W^−1^ in the visible wavelength, rivaling the room-temperature performance of the typical photodetectors.

## Results

### Properties of Ta_2_NiSe_5_ in EI phase

**Fig. 1:**
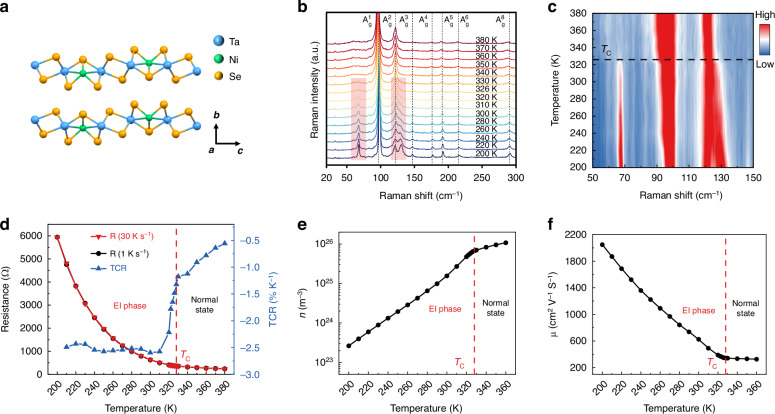
**EI phase in Ta**_**2**_**NiSe**_**5**_ **and its properties. a** Crystal structure of Ta_2_NiSe_5_. **b** Variable temperature Raman spectra of Ta_2_NiSe_5_. **c** Raman mapping of the new peaks. **d** Temperature-dependent resistance and TCR of Ta_2_NiSe_5_ nanosheet at two different cooling rates. **e** Carrier concentration of Ta_2_NiSe_5_ nanosheet. **f** Hall mobility of Ta_2_NiSe_5_ nanosheet

Ta_2_NiSe_5_ crystallizes in a quasi-one-dimensional structure, consisting of alternating layers of TaSe_2_ and NiSe_2_ along the c-axis. Each layer comprises hexagonal coordination of transition metal atoms sandwiched between Se atoms (Fig. 1a). We firstly utilized variable-temperature micro-Raman spectroscopy to conduct a characterization of high-quality Ta_2_NiSe_5_ nanosheet obtained through mechanical exfoliation in the temperature range of 200 K–380 K. The specific findings are elucidated in Fig. 1b. Above *T*_C_ of 326 K, seven distinct Raman characteristic peaks, situated at 97.2, 121.3, 146.9, 175.2, 190.6, 213.8, and 288.2 cm^−^^1^, correspondingly associated with the Raman vibrational modes from ${A}_{g}^{1}$ to ${A}_{g}^{6}$ and ${A}_{g}^{8}$, are identified. Below *T*_C_, the Raman mapping diagram (Fig. 1c) unveils the emergence of two pronounced additional Raman peaks at 68.1 and 130.2 cm^−^^1^. Concurrently, a narrowing of the ${A}_{g}^{1}$ Raman characteristic peak is observed. These experimental results align harmoniously with the outcomes derived from first-principles calculations^32^, signifying the transition of Ta_2_NiSe_5_ from an orthorhombic crystal system to a monoclinic one. The crystallographic phase transition exerts a significant influence on the electrical properties. In subsequent variable-temperature resistance characterization, Ta_2_NiSe_5_ exhibits manifest property variations at *T*_C_. We subjected Ta_2_NiSe_5_ nanosheets to two distinct cooling rates, namely rapid (30 K s^−^^1^) and slow (1 K s^−^^1^), as depicted in Fig. 1d. It is evident that the two variable-temperature resistance curves nearly overlap, affirming that the distinct cooling processes do not affect the EI phase transition of Ta_2_NiSe_5_. Ta_2_NiSe_5_ demonstrates metallic behavior during the cooling process from 380 K to *T*_C_, while the temperature coefficient of resistance (TCR) *α* of Ta_2_NiSe_5_ exhibits a sudden transformation from −1.3% to −2.5% after the EI phase transition, accompanied by a noticeable transition in electrical resistance towards semi-conductivity. A discernible shift in transport properties of Ta_2_NiSe_5_ is noted before and after the phase transition through analyzing the variable-temperature resistance, thus we fabricated a six-terminal Hall standard target on Ta_2_NiSe_5_ nanosheets to carry out variable-temperature Hall characterization. During the cooling process, the Hall coefficient remains negative (Fig. S1a), which means Ta_2_NiSe_5_ consistently maintained its n-type behavior before and after the EI phase transition. The Hall coefficient exhibited a marked increase following *T*_C_ (Fig. S1b), giving rise to a drastic reduction from 1.1 × 10^26^ m^−^^3^ to 2.6 × 10^23^ m^−3^ in carrier concentration $n$ and a rapid enhancement in mobility $\mu$ from 327.6 cm^2^V^−^^1^s^−^^1^ to 2049.0 cm^2^V^−^^1^s^−1^ (Fig. 1e, f). The distinct properties exhibited by Ta_2_NiSe_5_ via EI phase transition undoubtedly promise to unveil novel phenomena in the realm of photoelectric detection.

### Temperature-dependent response of Ta_2_NiSe_5_ photodetector

**Fig. 2:**
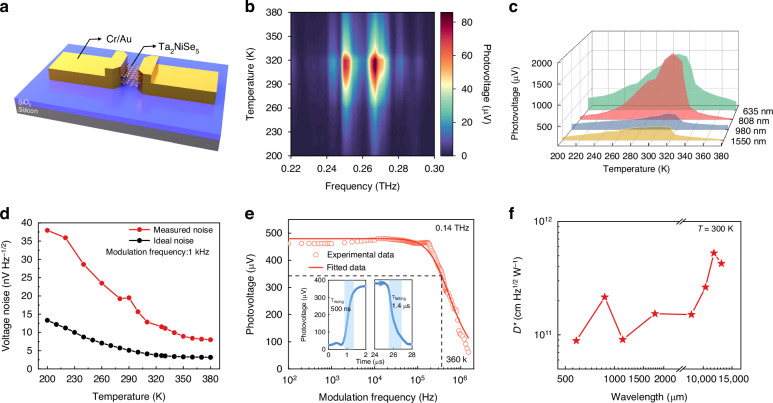
**Characterization of the temperature-dependent photoelectric performance of the Ta**_**2**_**NiSe**_**5**_ **device. a** Schematic structure of the Ta_2_NiSe_5_ device. **b** Temperature-dependent photovoltage of the device under irradiation in the 0.22–0.30 THz wave range. **c** Temperature-dependent photovoltage of the device under laser irradiation at 635, 808, 980, and 1550 nm. **d** Comparison between ideal and experimental temperature-dependent noise of the device at a modulation frequency of 1 kHz. **e** Experimental and fitted photovoltage as a function of the modulated frequency under 0.14 THz radiation. Inset: time-resolved photovoltage of the device at 0.14 THz. **f** Under ambient operating conditions, the optimal D^*^ values of the Ta_2_NiSe_5_ device in various terahertz wave ranges

We transferred Ta_2_NiSe_5_ nanosheets onto high-resistance silicon wafers coated with SiO_2_ and fabricated Cr/Au electrodes on the nanosheets. The channel gap and width between the electrodes are set at 2 μm and 5 μm, respectively. The thickness of the nanosheet is 20 nm (Fig. S2a). The schematic structure of the Ta_2_NiSe_5_ device is illustrated in Fig. 2a. For the terahertz frequency range, when low-energy terahertz photons are incident on the designed sub-wavelength structure, the electrons from the metal electrodes will be injected and trapped in the induced well located at the material. The conductivity of the material will be consequently changed and photocurrent signal can be collected between the metallic contacts^33,34^. The temperature-dependent photoelectric response spectra in the 0.22–0.30 THz range exhibit obvious photovoltage peaks within the room temperature range following the EI phase transition (Fig. 2b). The response of the photodetector aligns well under rapid (30 K s^−1^) and slow (1 K s^−1^) cooling rates, demonstrating its stability during the temperature variation process (Fig. S2b, c). The variable-temperature photoelectric response characterization results of the Ta_2_NiSe_5_ device at frequencies ranging from 0.020 to 0.035 THz, 0.14 THz, and 0.165 to 0.173 THz are presented in Fig. S2b–e, where the photovoltages uniformly exhibit notable peaks in the vicinity of room temperature following the EI phase transition. Simultaneously, the characterization of the terahertz band photovoltage for devices with thicknesses of 10, 18, 26, and 28 nm has presented curves consistent with the aforementioned outcomes, thus demonstrating the reproducibility of these results (Figs. S3 and S4). Regarding the VIS, NIR, and SWIR wavelength, where the photon energy exceeds the bandgap energy, the electrons in the valence band or impurity bands will be excited to the conduction band, forming nonequilibrium electron-hole pairs. The electrons and holes can be collected by the electrodes under applied electric field. Closely resembling the terahertz band, the Ta_2_NiSe_5_ device also exhibits analogous room-temperature photoelectric voltage peaks for wavelengths of 635, 808, 980, and 1550 nm (Fig. 2c).

In its high-temperature phase, Ta_2_NiSe_5_ behaves as a zero-gap semimetal. As the temperature decreases to *T*_C_, coulomb interactions between electrons and holes can result in spontaneous pairing correlations, leading to a new ground-state with BEC that exhibits macroscopic phase coherence with an associated interaction-induced insulating gap^35–37^. The excitonic condensation in EI phase results in a substantial reduction in carrier concentration, accompanied by enhanced mobility ascribing to decreased carrier scattering rate. These two aspects are crucial for improving the photoelectric response, as the theoretical deductions indicate that the photoelectric response is directly proportional to the mobility and inversely proportional to the carrier concentration. Simultaneously, owing to the unique EI phase transition temperature of Ta_2_NiSe_5_, transport properties including *α* and *μ* exhibit their maximum rates of change within the room temperature range. This results in an ambient peak in the photocurrent, enabling the achievement of photoelectric detection with room temperature extremum (Note S1). The noise voltage of the device increases with decreasing temperature, where the noise near room temperature closely approximates that at elevated temperatures, as depicted in Figs. 2d and S5. Concurrently, the response time of the device decreases with lowering temperature. However, the descent trend is somewhat attenuated post the EI phase transition, with no clear difference in response time between room temperature and the lower temperature of 200 K (Fig. S6). The cumulative experimental results above indicate that room temperature is the most optimal operational temperature for Ta_2_NiSe_5_ devices. The room-temperature photovoltage of the Ta_2_NiSe_5_ device exhibits a well-defined linear relationship with bias voltage and received radiation power (Fig. S7). Based on the optimized ambient photoelectric performance in Ta_2_NiSe_5_ device, we further conducted the detection of lower-energy photons in the frequency ranges of 0.340–0.346 THz and 0.508–0.519 THz at room temperature (Fig. S8). Electrical bandwidth is a critical performance of the photodetector, which can significantly influence the scope of its applications. Therefore, a −3 dB frequency response under a modulated 0.14 THz source was measured at room temperature, the electrical bandwidth in terahertz waveband was obtained as 360 kHz (Fig. 2e). The time-resolved photovoltage is shown in the inset of Fig. 2e, the rising time (τ_rising_) and falling time (τ_falling_) are estimated to be 500 ns and 1.4 μs, respectively. We further fit the −3 dB frequency photovoltage by the formula ${V}_{{ph}}=\frac{C}{\sqrt{1+{(2\pi \tau f)}^{2}}}$, where *V*_ph_ is the photovoltage, *τ* is the time constant obtained by fitting result, *C* is the constant, and *f* is the modulation frequency. Here, we obtain the *τ* to be 447 ns, which is consistent with the measured rising time (500 ns). We also recorded the waveform of photovoltage under *f* of 1, 50, and 200 kHz, showing that the output response kept square waveform at 0.14 THz when *f* < 200 kHz (Fig. S9). Regarding to the VIS, NIR and SWIR wavelengths, the photodetector exhibits electrical bandwidth of 19, 6, 5, and 18 kHz at 635, 808, 980, and 1550 nm, respectively (Fig. S10). The time constant at 635, 808, 980, and 1550 nm can be estimated as 8.4, 26.5, 31.8, and 8.8 μs, respectively. The rapid response speed of our photodetector across a broad band from visible and infrared wavelengths to terahertz waveband is sufficient to meet the requirement for environmental and remote sensing applications.

Responsivity is another crucial aspect for evaluating the performance of a photodetector. For photoconductive devices, the current responsivity is commonly used for characterization, whose formula is described as ${R}_{I}=\frac{{I}_{{ph}}}{{P}_{{in}}\times A}$, where *I*_ph_ is the photocurrent, *P*_in_ is the incident radiation power density and *A* is the effective area of the detector. At wavelengths of 635, 808, 980, and 1550 nm, the Ta_2_NiSe_5_ photodetector achieved peak ${R}_{I}$ at room temperature, with values of 25.3 A∙W^−1^, 4.1 A∙W^−1^, 1.1 A∙W^−^^1^, and 1.0 A∙W^−1^, respectively. In the terahertz band, the device room-temperature optimized ${R}_{I}$ at 0.169, 0.267, 0.344, and 0.513 THz, with values of 3.3 × 10^3^ A∙W^−1^, 2.0 × 10^3^ A∙W^−1^, 4.7 × 10^3^ A∙W^−^^1^, and 1.9 × 10^3^A∙W^−1^, respectively. Specific detectivity (D^*^), measuring the sensitivity of a photodetector normalized to its area and electrical bandwidth, is significant to assess the performance of the devices in practice. The D^*^ values of the Ta_2_NiSe_5_ device could be calculated using the formula ${D}^{* }=\frac{{V}_{{ph}}\sqrt{\Delta f}}{{P}_{{in}}{v}_{n}\sqrt{A}}$, where *v*_n_ is the noise voltage and Δ*f* is the bandwidth^38^. The D^*^ of Ta_2_NiSe_5_ photodetector at wavelengths of 635, 808, 980, and 1550 nm optimized at room temperature, with values of 7.8 × 10^8^ cm·Hz^1/2^·W^−^^1^, 1.2 × 10^8^ cm·Hz^1/2^·W^−^^1^, 3.1 × 10^7^ cm·Hz^1/2^·W^−^^1^, and 2.9 × 10^7^ cm·Hz^1/2^·W^−^^1^, respectively (Fig. S11a). Regarding to terahertz band, the obtained room-temperature D^*^ values as a function of wavelength are presented in Fig. S11b–f. Figure. 2f illustrates the response wavelength range and corresponding D^*^ values of Ta_2_NiSe_5_ device in the ultra-wide terahertz band, achieving optimal performance at 584, 870, 1130, 1770, and 11,600 μm, with superlative sensitivities of 8.9 × 10^10^ cm·Hz^1/2^·W^−1^, 2.2 × 10^11^ cm·Hz^1/2^·W^−^^1^, 7.3 × 10^10^ cm·Hz^1/2^·W^−^^1^, 1.5 × 10^11^ cm·Hz^1/2^·W^−1^, and 5.3 × 10^11^ cm·Hz^1/2^·W^−^^1^, respectively. The extreme sensitivity of Ta_2_NiSe_5_ device exhibits a two-order-of-magnitude enhancement compared to the commercial room temperature terahertz device (Golay Detectors. https://www.tydexoptics.com/pdf/Golay_Detectors.pdf). Therefore, we achieve highly sensitive photoelectric detection optimized at room temperature in Ta_2_NiSe_5_ due to its unique EI phase transition, proving it to be a promising material ideally suitable for room temperature photodetection.

### Ta_2_NiSe_5_-WS_2_ vdW heterojunction photodetectors

While Ta_2_NiSe_5_ has demonstrated excellent room-temperature detection performance in the wide terahertz band, its response in the visible and infrared wavelength is not as impressive, the maximal D^*^ value achieves at 635 nm is merely 7.8 × 10^8^ cm·Hz^1/2^·W^−^^1^. In addition, the inherently narrow bandgap of Ta_2_NiSe_5_ after EI phase transition contributes to its relatively high dark current noise. One possible approach to solve these problems is to construct a heterojunction, where a potential barrier is formed at the junction interface, impeding the transport of charge carriers and thereby mitigating the levels of dark current and noise in the device. Furthermore, owing to the strong interlayer coupling, the heterojunction can manifest new physical and optical properties within its confines. The built-in electric field within the heterojunction accelerates the separation of photogenerated charge carriers under illumination, enhancing the detection capabilities of the device. Therefore, we have fabricated a four-terminal field-effect transistor device based on the Ta_2_NiSe_5_-WS_2_ vdW heterojunction to further explore high-performance, broadband room-temperature detection.

**Fig. 3:**
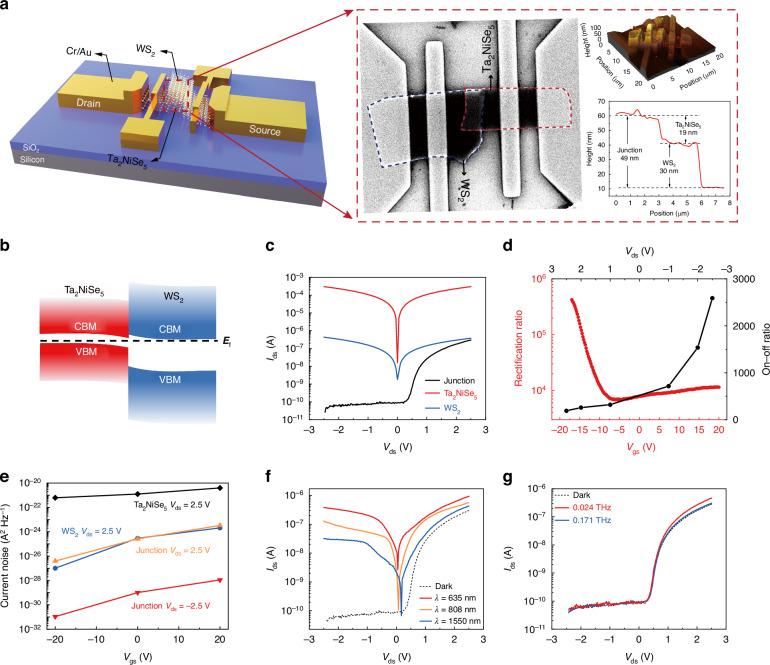
**Electrical characterization of heterojunction FET devices. a** Schematic diagram of the four-electrode Ta_2_NiSe_5_-WS_2_ vdW heterojunction FET device. Inset: SEM image of the device channel (left). AFM profile and AFM thickness curve of the device (right). **b** Schematic diagram of the band structure of Ta_2_NiSe_5_ and WS_2_ after contact. **c** IV curve of the source-drain electrode of each device in the four-electrode device. **d** On-off ratio curve and rectification ratio curve of the heterojunction device. **e** Experimental measured noise current of the devices at different gate voltages. **f** IV curves of the source-drain electrode of the heterojunction device under no light exposure and illumination with lasers at 635, 808, and 1550 nm. **g** IV curves of the source-drain electrode of the heterojunction device under no light exposure and with radiation at 0.024 THz and 0.171 THz

Figure. 3a illustrates a schematic diagram of the four-terminal field-effect transistor (FET) device. The respective control of the Ta_2_NiSe_5_, WS_2_, and heterojunction FET devices is achieved by applying voltage to the silicon back gate and selecting different source and drain electrodes. Fig. S12a showcases optical microscopic images of the device channels, with channel widths of 2 μm for Ta_2_NiSe_5_ and WS_2_ FETs, and 6 μm for the heterojunction FET. Scanning electron microscope (SEM) and atomic force microscope (AFM) characterizations were conducted to validate the efficacy of the heterojunction transfer. SEM images reveal high-quality contact areas formed by the overlapping regions of the two-dimensional nanosheets, as clearly depicted in the AFM three-dimensional profiles (Fig. 3a). Furthermore, AFM characterizations disclose the thickness of detached Ta_2_NiSe_5_ and WS_2_ nanosheets, as well as the transferred heterojunction, measuring 19, 30, and 49 nm, respectively. Raman spectroscopy was employed to assess the quality of the heterojunction, obtaining Raman spectra under 532 nm laser excitation for Ta_2_NiSe_5_ and WS_2_ as single materials, as well as the heterojunction region. The spectra in the overlapping region simultaneously exhibit characteristic Raman peaks of both materials, affirming the high quality of the materials post-exfoliation and transfer (Fig. S12b).

The transfer characteristics measured under both positive and negative bias conditions exhibit typical n-type behavior for Ta_2_NiSe_5_ FET, WS_2_ FET, and heterojunction FET (Fig. S13a). The field-effect mobility of Ta_2_NiSe_5_ and WS_2_, as inferred from these curves, measure as 556.5 cm^2^∙V^−^^1^∙s^−^^1^, and 1.8 cm∙V^−^^1^∙s^−1^, respectively (Fig. S13b, c). The carrier concentrations for Ta_2_NiSe_5_ and WS_2_ are 1.08 × 10^17^ cm^−^^3^ and 7.02 × 10^16^ cm^−^^3^, respectively. Consequently, the Fermi level difference between Ta_2_NiSe_5_ and WS_2_ is estimated at 0.08 eV and 0.11 eV (Note S2). Infrared ellipsometry and Kelvin probe force microscopy characterizations were conducted for an accurate assessment of the heterojunction band structure. Fitting the data with Lorentzian and Gaussian oscillator models yield bandgaps of 0.363 eV and 1.519 eV for Ta_2_NiSe_5_ and WS_2_, respectively (Fig. S14). The work functions for Ta_2_NiSe_5_ and WS_2_ are determined to be 4.67 eV and 4.49 eV, with their Fermi levels position 0.28 eV above the valence band and 1.41 eV above the valence band, respectively (Fig. S15). Based on the analysis above, the band structure distribution of Ta_2_NiSe_5_ and WS_2_ prior to contact has been inferred (Fig. S16). Figure. 3b illustrates a schematic diagram of the band structure distribution and changes after contact between the two materials. The Fermi level of WS_2_ is higher than that of Ta_2_NiSe_5_ prior to contact, therefore, Ta_2_NiSe_5_ and WS_2_ form a Type I n-n^+^ junction with electrons flowing from WS_2_ to Ta_2_NiSe_5_ upon contact, causing an upward bending of the Ta_2_NiSe_5_ band and a downward bending of the WS_2_ band. Figure. 3c depicts the source-drain current (*I*_ds_) and source-drain voltage (*V*_ds_) curves for Ta_2_NiSe_5_, WS_2_, and heterojunction FETs under zero gate voltage. At a bias voltage of −2.5 V, the dark current of the heterojunction device decreases by seven orders of magnitude in comparison to the Ta_2_NiSe_5_ device and five orders of magnitude compared to the WS_2_ device. Owing to the high mobility of Ta_2_NiSe_5_, gate voltage does not significantly affect the channel of its FET device, while the introduction of WS_2_ significantly enhances the gate voltage controllability of the heterojunction FET channel. The gate control curves under different biases are illustrated in Fig. S17. The heterojunction FET device exhibits an on-off ratio of 2600 at *V*_ds_ = 2.5 V and a rectification ratio of 4 × 10^5^ at *V*_gs_ = −20 V as depicted in Fig. 3d, highlighting its capability to create high depletion region barriers and voltage control. Moreover, gate voltage control substantially reduces the noise current of device. The noise of photodetectors is primarily induced by thermal noise, shot noise and 1/*f* noise. The devices were measured at a modulation frequency of 1 kHz, thus the 1/*f* noise could be ignored as it only exists at low frequency. Hence, the theoretical current noise could be calculated as ${i}_{n}=\sqrt{\frac{4{k}_{B}T\Delta f}{R}+2e{I}_{d}\Delta f}$,where *e* is the unit electric charge, *R* is the resistance at bias and *I*_d_ is the dark current^39^. Under *V*_gs_ of −20 V, the measured noise current of the heterojunction FET device experiences a three-order-of-magnitude reduction under reverse biases and a two-order-of-magnitude reduction under forward biases (Fig. 3e), consistent with the theoretical calculations (Fig. S18). Subsequently, we characterized the photoelectric response of the heterojunction device at room temperature, whose IV curves exhibit a notable photovoltaic effect in the form of photocurrent under the illumination of lasers at 635, 808, and 1550 nm, as displayed in Fig. 3f. The IV curves of the heterojunction devices under conditions of terahertz radiation and no illumination are illustrated in Fig. 3g, where the device demonstrates significant photocurrents at 0.024 THz and 0.171 THz under forward bias.

**Fig. 4:**
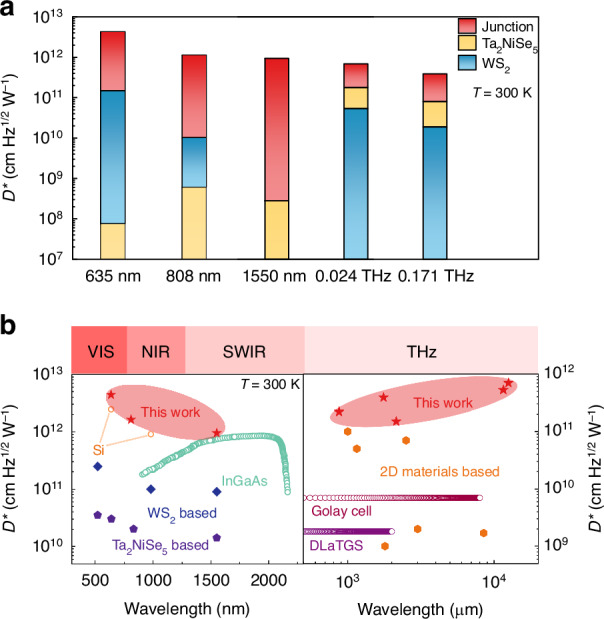
**Characterization and comparison of the photoelectric performance of the heterojunction device. a** Photoelectric performance comparison of each device in the four-electrode device across different wavelength ranges. **b** Performance comparison of Ta_2_NiSe_5_-based photodetectors with commercial photodetectors and reported 2D material photodetectors

The specific detectivity D^*^ values of the vdW heterojunction device could be calculated using the formula ${D}^{* }=\frac{{I}_{{ph}}/{P}_{{in}}}{\sqrt{\frac{4{k}_{B}{TA}}{R}+2e{I}_{d}A}}$. Figure. 4a provides a detailed comparison of the D^*^ values for each device in the four-electrode device across five different frequency bands. At 0.024 THz and 0.171 THz, the heterojunction device achieves maximum D^*^ values of 7.0 × 10^11^ cm·Hz^1/2^·W^−^^1^ and 3.9 × 10^11^ cm·Hz^1/2^·W^−^^1^ at *V*_ds_ = 2.5 V, respectively, which represents an order of magnitude improvement compared to the WS_2_ device and a fourfold increase compared to the Ta_2_NiSe_5_ device (Figs. 4a and S19a). The terahertz responses of all three devices exhibit a clear power-linear relationship (Fig. S19b). Under forward bias, the electrodes efficiently collect electrons injected from the metal into the material under the influence of the EIW effect^33,34^. However, under reverse bias, the device does not respond, as the high conduction band barrier impedes the flow of electrons (Fig. S20). The response spectra of Ta_2_NiSe_5_, WS_2_, and heterojunction devices in the frequency ranges of 0.02–0.04 THz and 0.163–0.173 THz are depicted in Fig. S19c, d, respectively, demonstrating that the response of the heterojunction device arises from the collaborative interaction of the materials at both ends of the junction. The response time of the heterojunction device has been improved to 5 μs, compared to the response speeds of Ta_2_NiSe_5_ and WS_2_ devices (Figs. S21 and S22). Given the inherently high sensitivity of the Ta_2_NiSe_5_ itself in the terahertz band, the performance improvement of the heterojunction device is not significant. However, for the VIS, NIR, and SWIR wavelengths where Ta_2_NiSe_5_ exhibits a weaker intrinsic response, the heterojunction device demonstrates a notable enhancement in performance. In the 635 nm and 808 nm wavelength where the photon energy exceeds the bandgap widths of both Ta_2_NiSe_5_ and WS_2_, charge carriers can be collected by the metal electrodes at both ends under both forward and reverse bias conditions due to the characteristics of the Type I heterojunction (Fig. S23a). Under laser illumination at 635 nm, the heterojunction FET device under reverse bias exhibits a maximum ${R}_{I}$ of 3.1 A∙W^−^^1^ and D^*^ of 4.1 × 10^12^ cm·Hz^1/2^·W^−1^ (*V*_gs_ = −17 V) with gate voltage modulation, representing an astonishing five orders of magnitude improvement compared to the Ta_2_NiSe_5_ device and an one order of magnitude improvement compared to the WS_2_ device. Under laser illumination at 808 nm, the heterojunction FET device under reverse bias achieves a maximum ${R}_{I}$ of 2.5 A ∙ W^−1^ and D^*^ of 1.2 × 10^12^ cm·Hz^1/2^·W^−1^ (*V*_gs_ = −17 V) with gate voltage modulation, which exhibits a three orders of magnitude improvement compared to the Ta_2_NiSe_5_ device and a two orders of magnitude improvement compared to the WS_2_ device (Figs. 4a and S24a, b). For the wavelength of 1550 nm, the photon energy is not sufficient to induce transitions in the valence band electrons of WS_2_ but still exceeds the bandgap width of Ta_2_NiSe_5_, thus the response of the heterojunction device is primarily attributed to the influence of Ta_2_NiSe_5_ (Fig. S23b). Under laser illumination at 1550 nm, the heterojunction FET device under reverse bias achieves a maximum ${R}_{I}$ of 0.7 A∙W^−^^1^ and D^*^ of 9.4 × 10^11^ cm·Hz^1/2^·W^−1^ (*V*_gs_ = −17 V) with gate voltage modulation. This not only represents a four orders of magnitude improvement compared to the Ta_2_NiSe_5_ device but also broadens the response band compared to WS_2_ device (Figs. 4a and S24c). The responses of all three devices in VIS, NIR and SWIR exhibit a linear relationship with power (Fig. S24d–f). In addition to the significant improvement in detection sensitivity, the heterojunction device also demonstrates a certain degree of enhancement in response time compared to Ta_2_NiSe_5_ and WS_2_ devices at these wavelengths (Figs. S25–S29). To further validate the performances including responsivity, electrical bandwidth and D^*^ of our devices, we compared them with other reported devices and some commercial devices in Figs. 4b and S30, whose detailed data is presented in Tables S1 and S2. In the terahertz band, our device exhibits greater ambient electrical bandwidth compared to commercial devices and 2D material photodetector, as is shown in Fig. S30a. Additionally, our device achieves a notable improvement of up to three-order-of-magnitude in ${R}_{I}$ and up to two-order-of-magnitude in D^*^ compared to commercial broadband terahertz devices like Golay Cell and DLaTGS that operate at room temperature. Furthermore, compared to terahertz devices based on two-dimensional materials reported in other researches, our device also shows higher responsivity and nearly one-order-of-magnitude enhancement in D^*^ (Table S1). In the VIS, NIR, and SWIR wavelengths, our device exhibits room-temperature D^*^ values superior to commercial InGaAs devices and comparable to D^*^ values measured for commercial Si devices under same experimental conditions, while the limited electrical bandwidth compared to commercial devices makes it more suitable for sensing and imaging applications in the infrared wavelength (Fig. S30b). Moreover, compared to the previously reported devices based on Ta_2_NiSe_5_ and WS_2_, our device demonstrates an enhancement in ${R}_{I}$ and electrical bandwidth, as well as an improvement of one to two-order-of-magnitude in D^*^ (Table S2). The numerous advantages outlined above substantiate the exceptional potential of our proposed EI-based detectors in infrared sensing, terahertz applications, and ultra-wideband detection.

## Discussion

We have successfully achieved room-temperature optimized photoelectric response with extreme sensitivity utilizing the EI transition in Ta_2_NiSe_5_ for the first time. High-performance, broadband photodetection has been demonstrated across VIS, NIR, SWIR, and THz spectra at room temperature using Ta_2_NiSe_5_ and its heterostructure. The Ta_2_NiSe_5_ photoconductor exhibits extraordinary sensitivity and an exceptionally broadband response in the terahertz wavelength range from 580 μm to 15,000 μm, achieving a maximal D^*^ of 5.3 × 10^11^ cm·Hz^1/2^·W^−^^1^, which represents a two-order-of-magnitude enhancement compared to commercial room-temperature terahertz detectors, such as Golay Cell and DLaTGS and nearly one-order-of-magnitude enhancement compared to the state-of-the-art room-temperature terahertz detectors. Additionally, it demonstrates outstanding bias linearity, power linearity, and exhibits electrical width of 360 kHz with a time constant of 500 ns. To suppress the dark current at room temperature, the vdW heterojunction detector has been fabricated by combining Ta_2_NiSe_5_ and WS_2_. The device exhibits excellent rectification of 3 × 10^5^ and on-off ratio of 2600, respectively. Compared to the photoconductor devices, the heterojunction photodetector expands the spectral range beyond the capability of WS_2_ and achieves a significant improvement in D^*^, reaching 4.1 × 10^12^ cm·Hz^1/2^·W^−^^1^, 1.2 × 10^12^ cm·Hz^1/2^·W^−^^1^ and 9.4 × 10^11^ cm·Hz^1/2^·W^−^^1^ at 635 nm, 808 nm, and 1550 nm, respectively. The heterojunction photodetector exhibits D* comparable to the room-temperature response of typical infrared detectors such as Si devices and InGaAs devices. Its photoelectric performance in the terahertz ranges with D^*^ value of 7.0 × 10^11^ cm·Hz^1/2^·W^−1^ has also shown further improvement compared to photoconductive device. The results of the optimized room temperature photoelectric detection via EI phase transition open up a new direction for the potential applications in sensitive environmental and remote sensing employing room-temperature, high-performance photodetectors.

## Materials and methods

### Device fabrication

2D Ta_2_NiSe_5_ and WS_2_ materials were mechanically exfoliated from high-quality bulk materials. For Ta_2_NiSe_5_ devices, Ta_2_NiSe_5_ nanosheets were transferred onto high-resistance silicon wafers with a 300 nm SiO_2_ layer using PDMS. Electrode patterns were prepared using ultraviolet lithography. For Ta_2_NiSe_5_-WS_2_ heterojunction FET devices, WS_2_ nanosheets were precisely transferred onto Ta_2_NiSe_5_ nanosheets on high-resistance silicon wafers with a 300 nm SiO_2_ layer using a 2D material transfer platform. Source and drain electrode patterns were fabricated using electron beam lithography. Subsequently, Cr/Au (15/75 nm) thin films were grown on the silicon wafer with electrode patterns using dual ion beam sputtering. After the lift-off process, Ta_2_NiSe_5_ devices and Ta_2_NiSe_5_-WS_2_ heterojunction FET devices were successfully fabricated.

### Characterization and measurements

The variable temperature Raman spectra were characterized using a Lab Ram HR800 through 532 nm laser excitation with a power of 1 mW. The AFM images of the samples were acquired from Bruker Dimension Edge. All variable temperature electronic and photoelectric measurements were carried out using an Oxford Instruments cryostat Optistat CF-V and an Oxford Instruments temperature controller UMC0015. The variable resistances were measured with a Keithley source measure unit 2612B. The variable temperature photoelectric performances were characterized with a LabView controlled system consisted of terahertz wave sources, VIS and IR solid-state laser, a Stanford Research lock-in amplifier SR830, a Stanford Research preamplifier SR570. All the waveforms are captured from a Teledyne LeCroy high-speed sampling oscilloscope 62Xi-A. The variable temperature noise spectra were captured through a Stanford Research network analyzer SR770.

## Supplementary information

Supplementary Information

## Data Availability

Data that support the findings of this study are available from the corresponding authors upon reasonable request.
