# Supplementary material for: Ultrasensitive photoelectric detection with room temperature extremum
Source: Light Sci Appl. 2025 Feb 26;14:96. doi: 10.1038/s41377-024-01701-0 (PMC11861971; doi:10.1038/s41377-024-01701-0)
Supplement: Supplementary file 1 — Supplementary Information [file 41377_2024_1701_MOESM1_ESM.docx]

**Supplementary Information For**

**Ultrasensitive photoelectric detection with room temperature extremum**

Tuntan Wu^1,2,3^, Yongzhen Li^1,3^, Qiangguo Zhou^1,3^, Qinxi Qiu^1^, Yanqing Gao^1^, Wei Zhou^1^, Niangjuan Yao^1^, Junhao Chu^1,3,4^, Zhiming Huang^*1,2,3,4,5^

1. State Key Laboratory of Infrared Physics, Shanghai Institute of Technical Physics, Chinese Academy of Sciences, Shanghai 200083, China

2. Hangzhou Institute for Advanced Study, University of Chinese Academy of Sciences, Hangzhou 310024, China

3. University of Chinese Academy of Sciences, Beijing 100049, China

4. Institute of Optoelectronics, Fudan University, Shanghai 200438, China

5. Key Laboratory of Space Active Optical-Electro Technology, Chinese Academy of Sciences, Shanghai 200083, China

*Corresponding author. Email: [zmhuang@mail.sitp.ac.cn](mailto:zmhuang@mail.sitp.ac.cn)

**Table of contents**

[Fig.S1 The variable temperature electrical transport characteristics of the Hall bar. 1](#_Toc187241114)

[Fig.S2 The AFM image of Ta_2_NiSe_5_ device and variable temperature photovoltage of Ta_2_NiSe_5_ device across various terahertz frequencies. 2](#_Toc187241115)

[Fig.S3 The AFM image of Ta_2_NiSe_5_ device, variable temperature resistance and the temperature-dependent terahertz response of supplementary Ta_2_NiSe_5_ device. 3](#_Toc187241116)

[Fig.S4 The AFM image of Ta_2_NiSe_5_ device, variable temperature resistance and the temperature-dependent terahertz response of different Ta_2_NiSe_5_ devices. 4](#_Toc187241117)

[Fig.S5 The variable temperature noise spectra of Ta_2_NiSe_5_ device. 5](#_Toc187241118)

[Fig.S6 The variable temperature response time of Ta_2_NiSe_5_ device at 0.14 THz. 6](#_Toc187241119)

[Fig.S7 The performance characterization of the Ta_2_NiSe_5_ device in the terahertz frequency range. 7](#_Toc187241120)

[Fig.S8 The room temperature photovoltage of Ta_2_NiSe_5_ device in terahertz frequency range. 8](#_Toc187241121)

[Fig.S9 The waveforms and electrical bandwidth in terahertz range at room temperature. 9](#_Toc187241122)

[Fig.S10 The ambient electrical bandwidth at VIS, NIR and SWIR wavelengths. 10](#_Toc187241123)

[Fig.S11 The room-temperature D^*^ values of Ta_2_NiSe_5_ device at different wavelengths. 11](#_Toc187241124)

[Fig.S12 The characterization of the Ta_2_NiSe_5_-WS_2_ vdW heterojunction. 12](#_Toc187241125)

[Fig.S13 Electrical characterization of the Ta_2_NiSe_5_-WS_2_ vdW heterojunction device. 13](#_Toc187241126)

[Fig.S14 Infrared spectroscopic ellipsometry (SE) characterization of Ta_2_NiSe_5_ and WS_2_. 14](#_Toc187241127)

[Fig.S15 Work function measurements of Ta_2_NiSe_5_ and WS_2_ by KPFM. 15](#_Toc187241128)

[Fig.S16 Energy band alignments of Ta_2_NiSe_5_-WS_2_ vdW heterojunction by SE and KPFM. 16](#_Toc187241129)

[Fig.S17 Transfer curves of Ta_2_NiSe_5_-WS_2_ heterojunction device under different bias voltages. 17](#_Toc187241130)

[Fig.S18 Noise characterization of the four-terminal device. 18](#_Toc187241131)

[Fig.S19 Performance characterization of four-terminal device in the terahertz frequency range. 19](#_Toc187241132)

[Fig.S20 Schematic illustration of the optoelectronic response of the Ta_2_NiSe_5_-WS_2_ vdW heterojunction device in the terahertz frequency range. 20](#_Toc187241133)

[Fig.S21 Response waveforms of individual devices within the four-terminal device at 0.024 THz. 21](#_Toc187241134)

[Fig.S22 Response time under variable gate voltage of individual devices within the four-terminal device at 0.024 THz. 22](#_Toc187241135)

[Fig.S23 Schematic illustration of the optoelectronic response of the Ta_2_NiSe_5_-WS_2_ vdW heterojunction device in the VIS, NIR and SWIR wavelength. 23](#_Toc187241136)

[Fig.S24 Performance characterization of individual devices within the four-terminal device in the VIS, NIR and SWIR wavelength. 24](#_Toc187241137)

[Fig.S25 Response waveforms of individual devices within the four-terminal device at 635 nm. 25](#_Toc187241138)

[Fig.S26 Response time under variable gate voltage of individual devices within the four-terminal device at 635 nm. 26](#_Toc187241139)

[Fig.S27 Response waveforms of individual devices within the four-terminal device at 808 nm. 27](#_Toc187241140)

[Fig.S28 Response time under variable gate voltage of individual devices within the four-terminal device at 808 nm. 28](#_Toc187241141)

[Fig.S29 Response waveforms and response time under variable gate voltage of individual devices within the four-terminal device at 1550 nm. 29](#_Toc187241142)

[Fig.S30 Performance comparison of our photodetectors with previous photodetectors at room temperature. 30](#_Toc187241143)

[Table S1. Comparison on photoelectric performances in terahertz regime among reported 2D materials photodetectors and commercial devices at room temperature. 31](#_Toc187241144)

[Table S2. Comparison on photoelectric performances in infrared regime among reported 2D materials photodetectors and commercial devices at room temperature. 32](#_Toc187241145)

[Note S1. Temperature-dependent photocurrent of Ta_2_NiSe_5_ 33](#_Toc187241146)

[Note S2. Band structure calculation of Ta_2_NiSe_5_ and WS_2_ 37](#_Toc187241147)

[Supplementary References 38](#_Toc187241148)

**
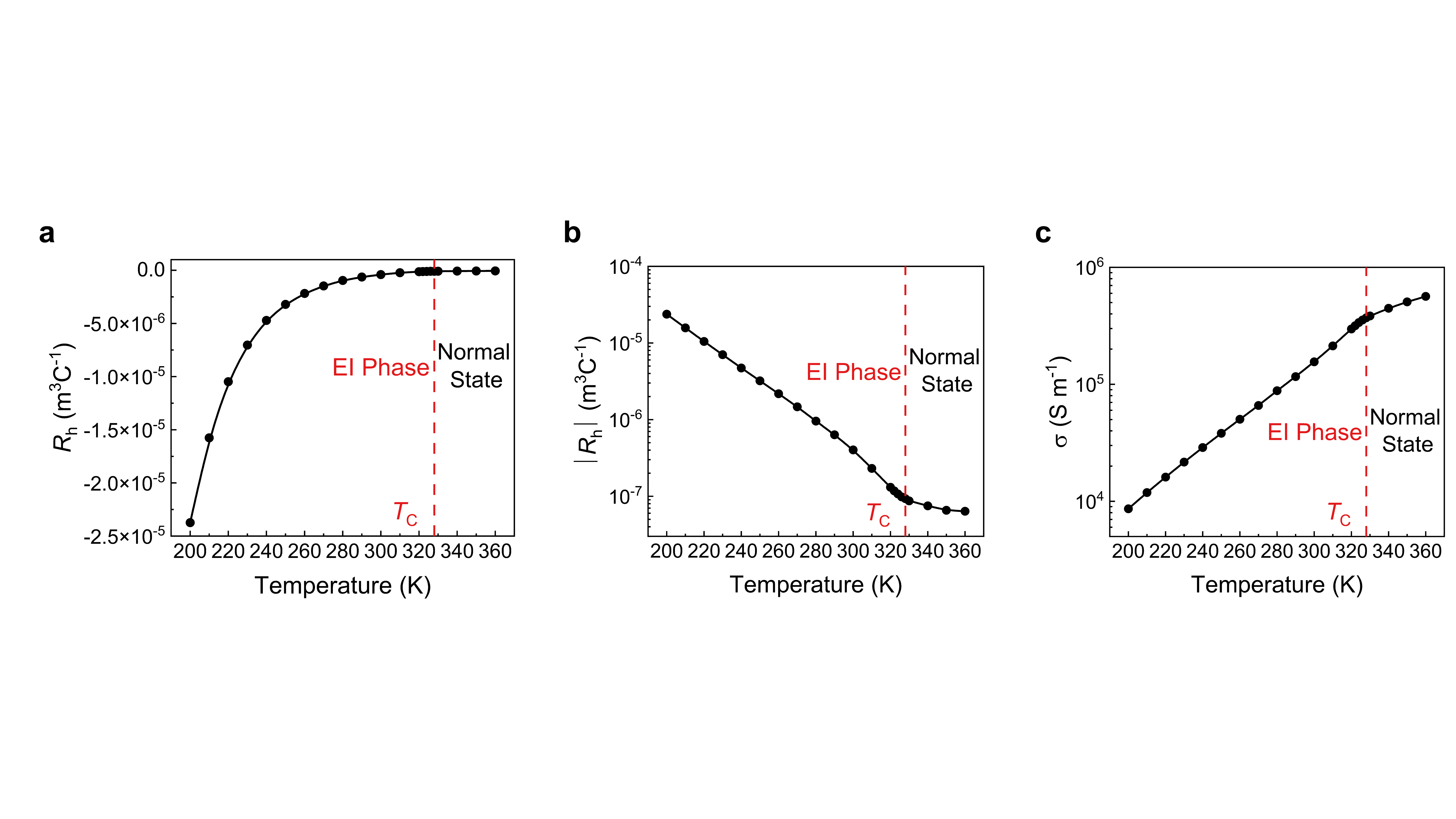
**

Fig.S1 The variable temperature electrical transport characteristics of the Hall bar. **a,** The variable temperature Hall coefficient of Ta_2_NiSe_5_ nanosheet (linear coordinate). **b,** The variable temperature Hall coefficient of Ta_2_NiSe_5_ nanosheet (exponential coordinate). **c,** The variable temperature electrical conductivity of Ta_2_NiSe_5_ nanosheet.

**
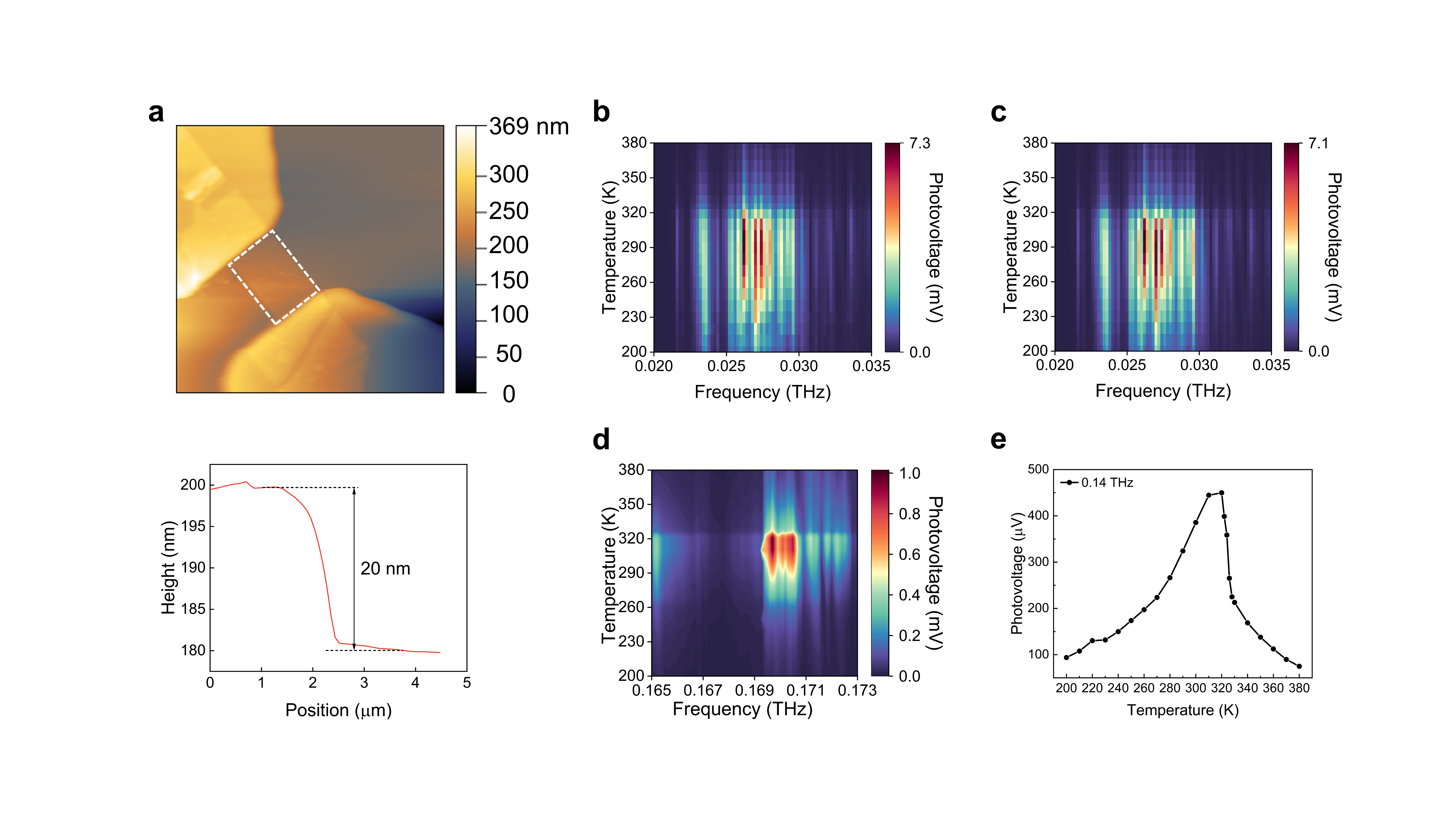
**

Fig.S2 The AFM image of Ta_2_NiSe_5_ device and variable temperature photovoltage of Ta_2_NiSe_5_ device across various terahertz frequencies. **a,** The AFM image of Ta_2_NiSe_5_ device**.** The thickness of Ta_2_NiSe_5_ sheet is 20 nm. **b,** Various temperature photovoltage at 0.020-0.035 THz under cooling rate of 30 K s^-1^. **c,** Various temperature photovoltage at 0.020-0.035 THz under cooling rate of 1 K s^-1^. **d,** Various temperature photovoltage at 0.165-0.173 THz. **e,** Various temperature photovoltage at 0.14 THz.

**
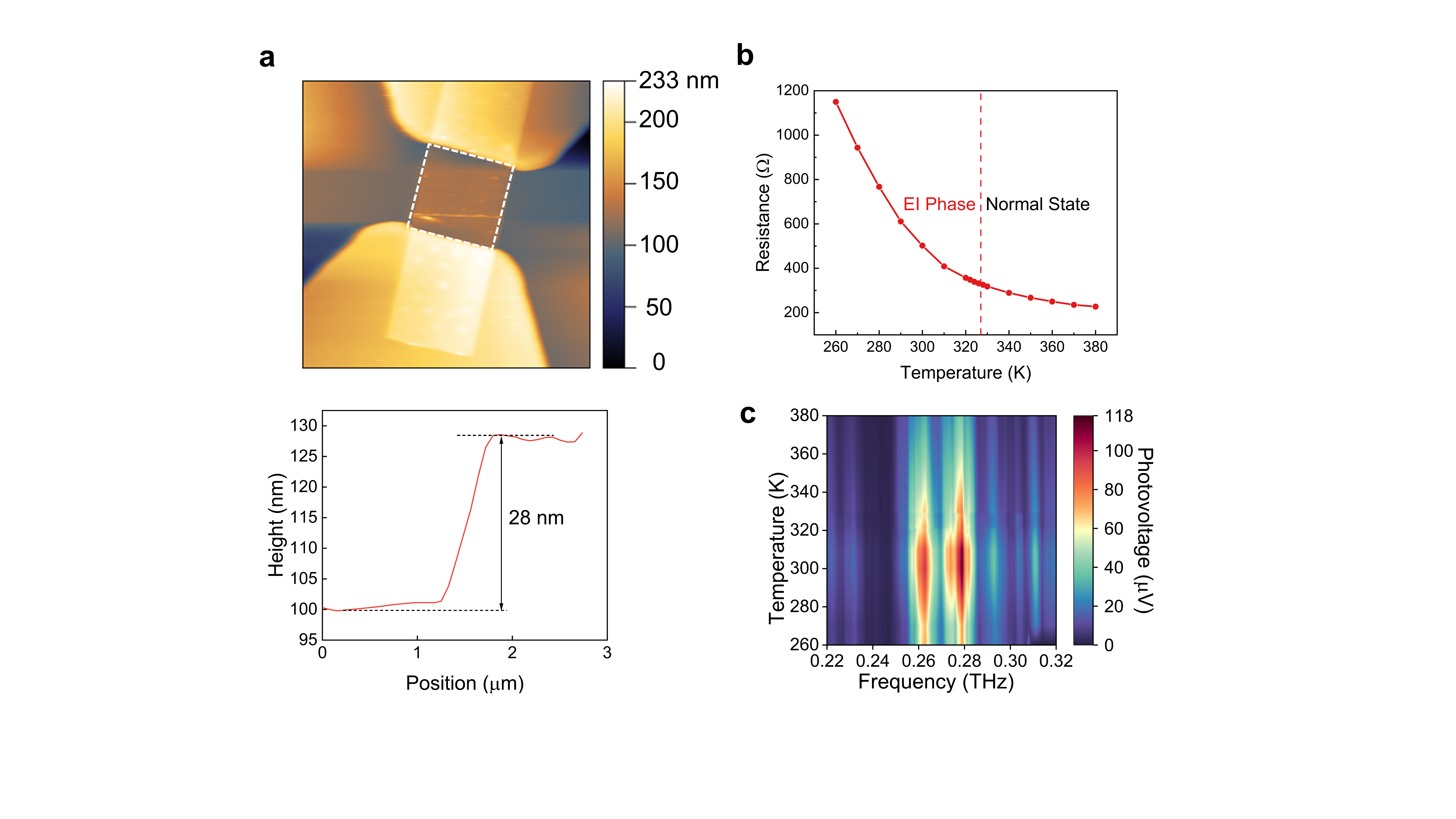
**

Fig.S3 The AFM image of Ta_2_NiSe_5_ device, variable temperature resistance and the temperature-dependent terahertz response of supplementary Ta_2_NiSe_5_ device. **a,** The AFM image of Ta_2_NiSe_5_ device**.** The thickness of Ta_2_NiSe_5_ sheet is 28 nm. **b,** The variable temperature resistance of Ta_2_NiSe_5_ device. **c,** The temperature-dependent photovoltage of Ta_2_NiSe_5_ device in the 0.22-0.32 THz frequency range.

**
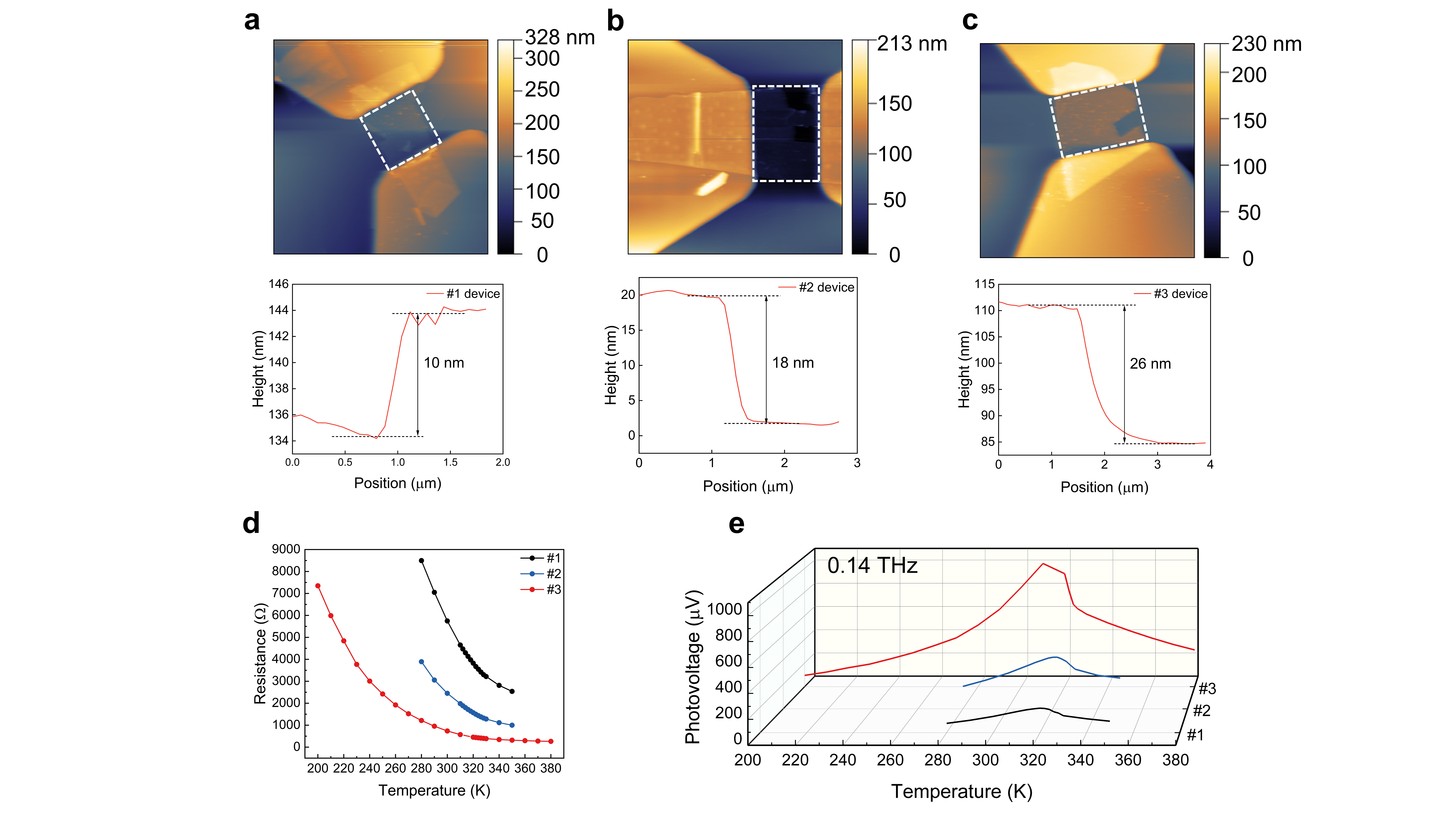
**

Fig.S4 The AFM image of Ta_2_NiSe_5_ device, variable temperature resistance and the temperature-dependent terahertz response of different Ta_2_NiSe_5_ devices. **a-c,** The AFM image of Ta_2_NiSe_5_ devices**.** The thickness of Ta_2_NiSe_5_ sheet is 10 nm, 18 nm and 26 nm, respectively. **d,** The variable temperature resistance of Ta_2_NiSe_5_ devices. **e,** The temperature-dependent photovoltage of three different Ta_2_NiSe_5_ devices at 0.14 THz.

**
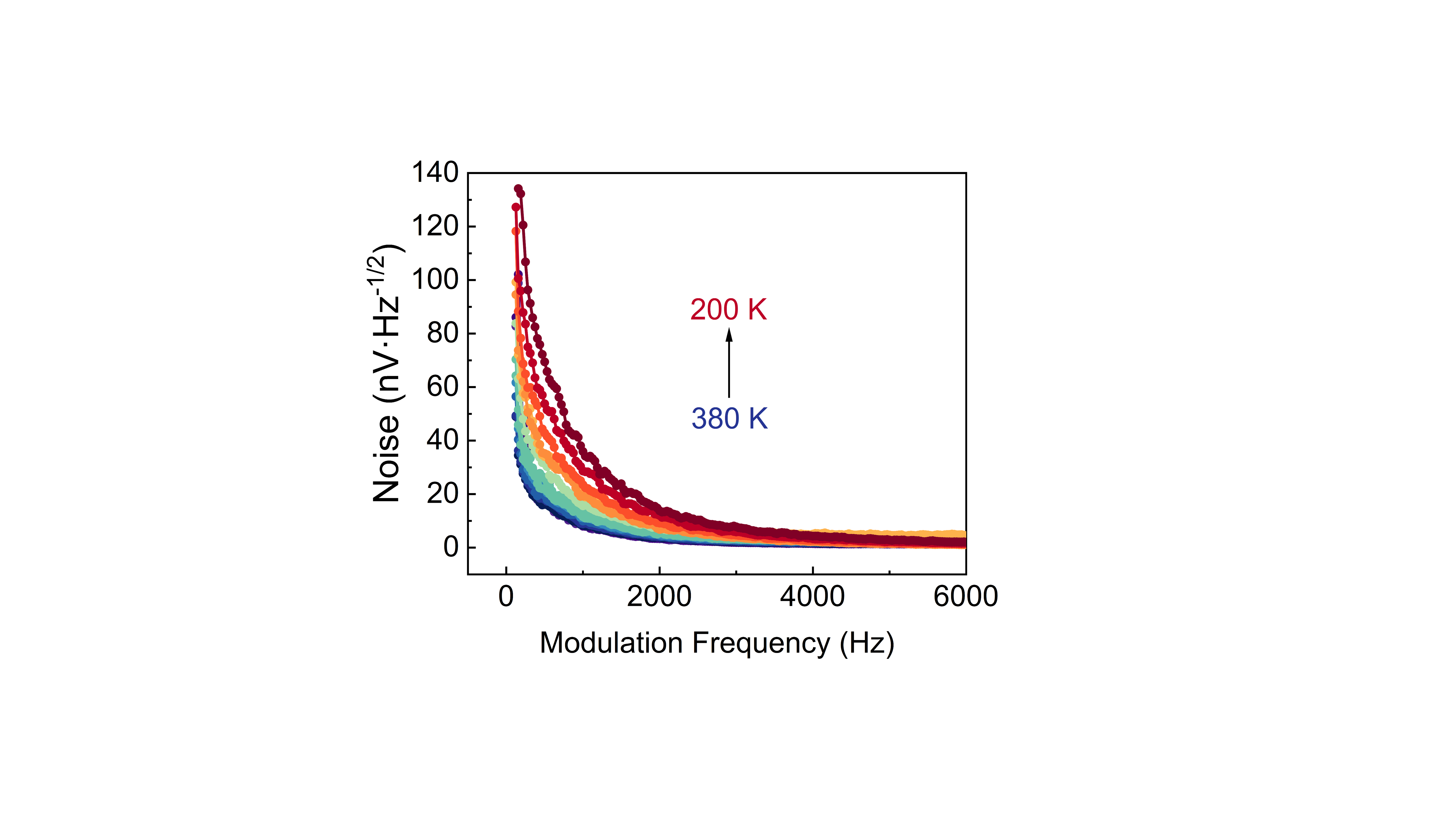
**

Fig.S5 The variable temperature noise spectra of Ta_2_NiSe_5_ device.

**
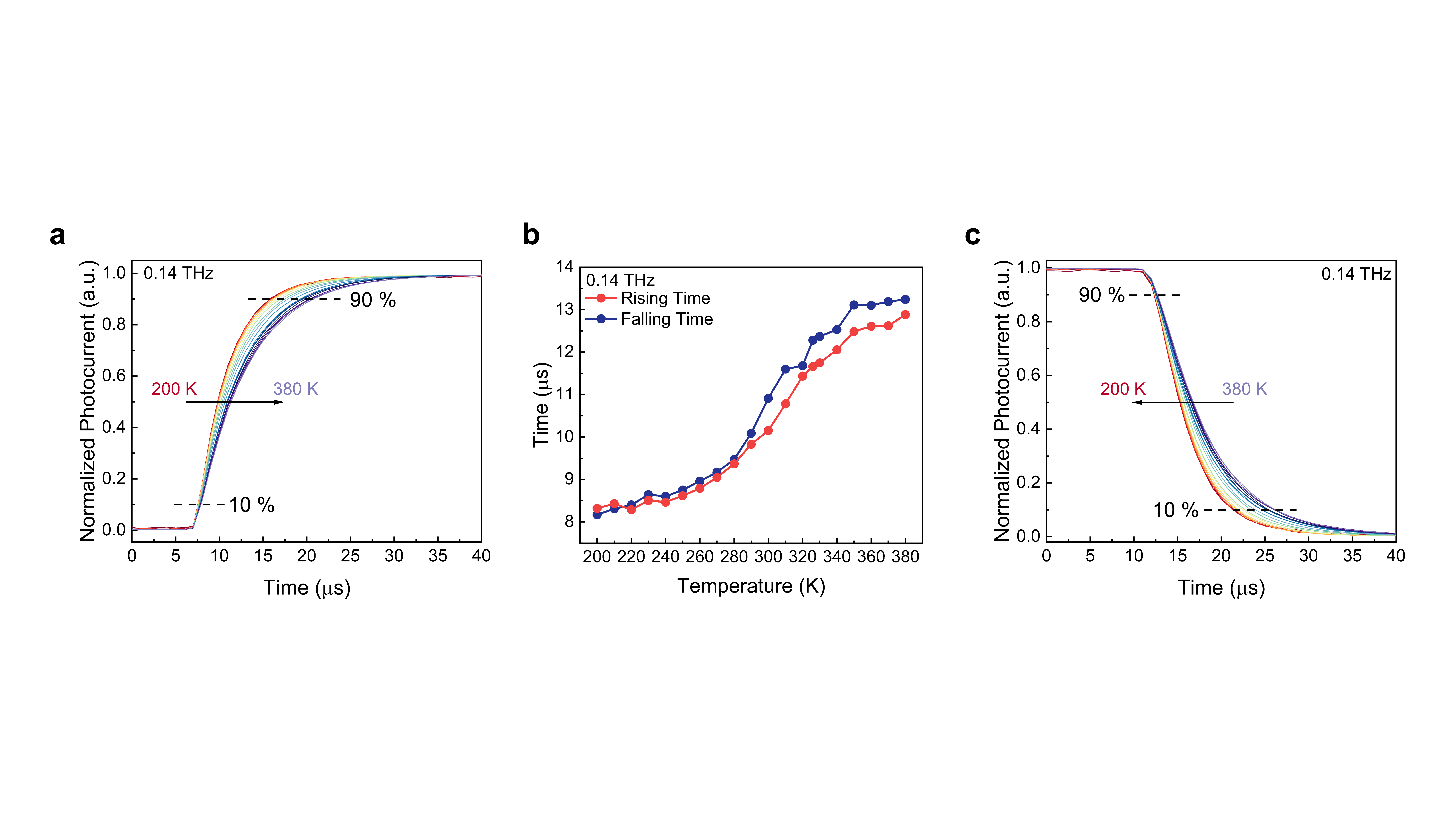
**

Fig.S6 The variable temperature response time of Ta_2_NiSe_5_ device at 0.14 THz. **a,** Rising time from 380 K to 200 K. **b,** Variable temperature rising time and falling time. **c,** Falling time from 380 K to 200 K.

**
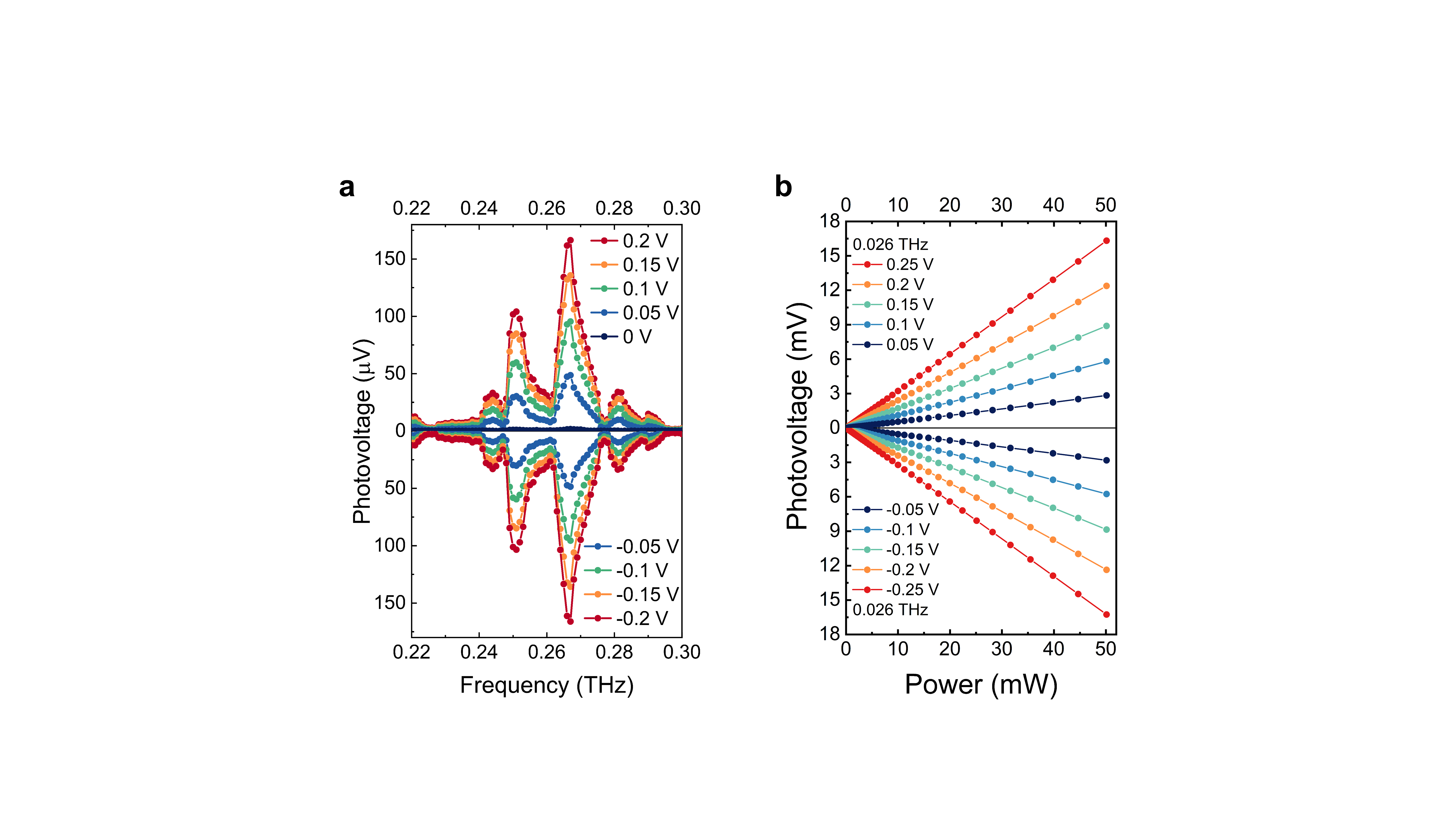
**

Fig.S7 The performance characterization of the Ta_2_NiSe_5_ device in the terahertz frequency range. **a,** Response spectra of the device at 0.22-0.3 THz with respect to bias. **b,** Photovoltages of the device at 0.026 THz with increasing bias and power.

**
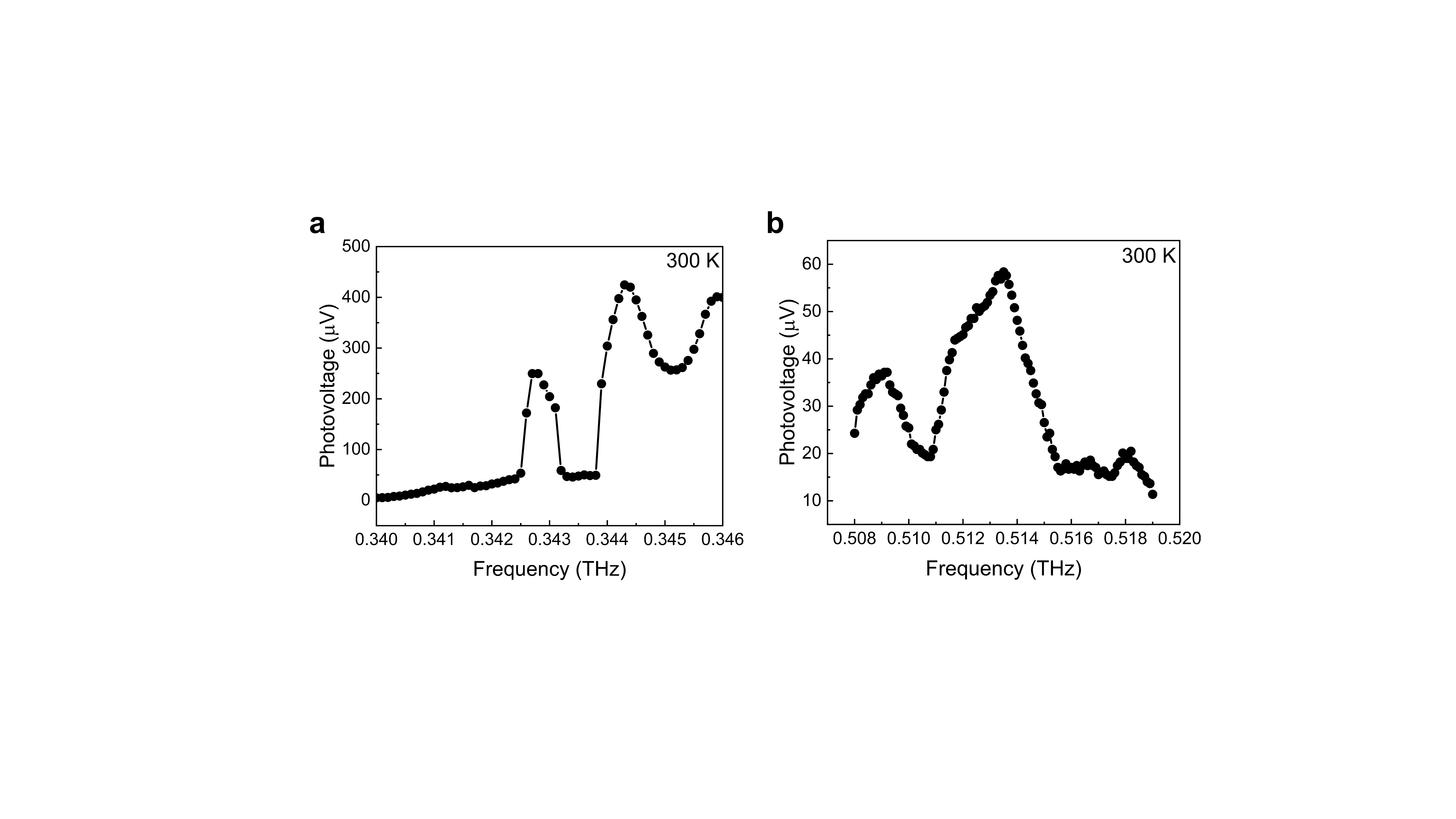
**

Fig.S8 The room temperature photovoltage of Ta_2_NiSe_5_ device in terahertz frequency range. **a,** 0.340-0.346 THz. **b,** 0.508-0.519 THz.


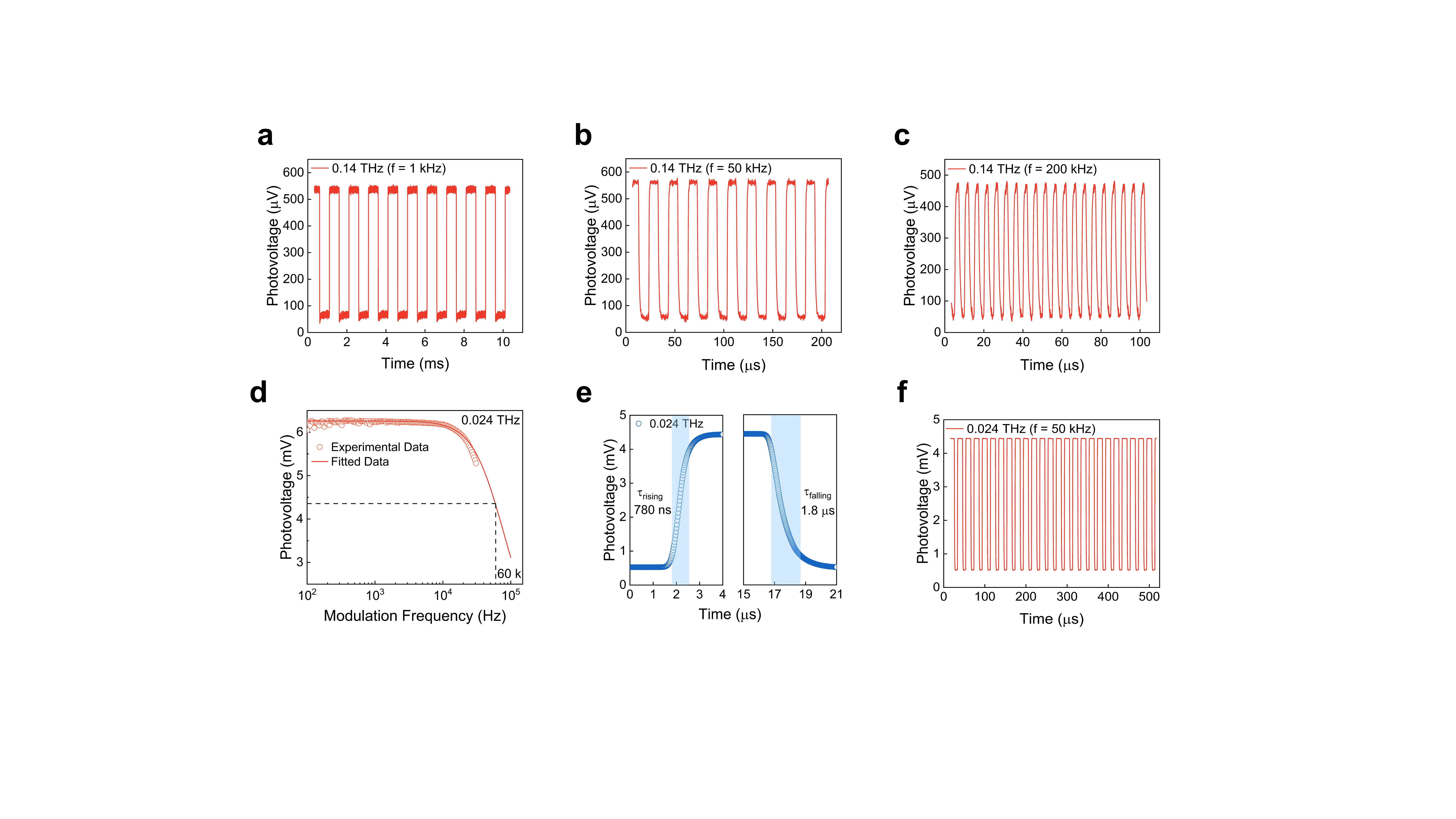


Fig.S9 The waveforms and electrical bandwidth in terahertz range at room temperature. **a-c,** The waveform at 0.14 THz under modulation frequency of 1 kHz, 50 kHz and 200 kHz. **d,** Photovoltage as a function of the modulation frequency under the radiation of 0.024 THz radiation. The -3 dB frequency is measured as 60 kHz. **e,** Time-resolved photovoltage at 0.024 THz. the τ_rising_ and τ_falling_ is 780 ns and 1.8 μs, respectively. **f,** The waveform at 0.024 THz under modulation frequency of 50 kHz.

**
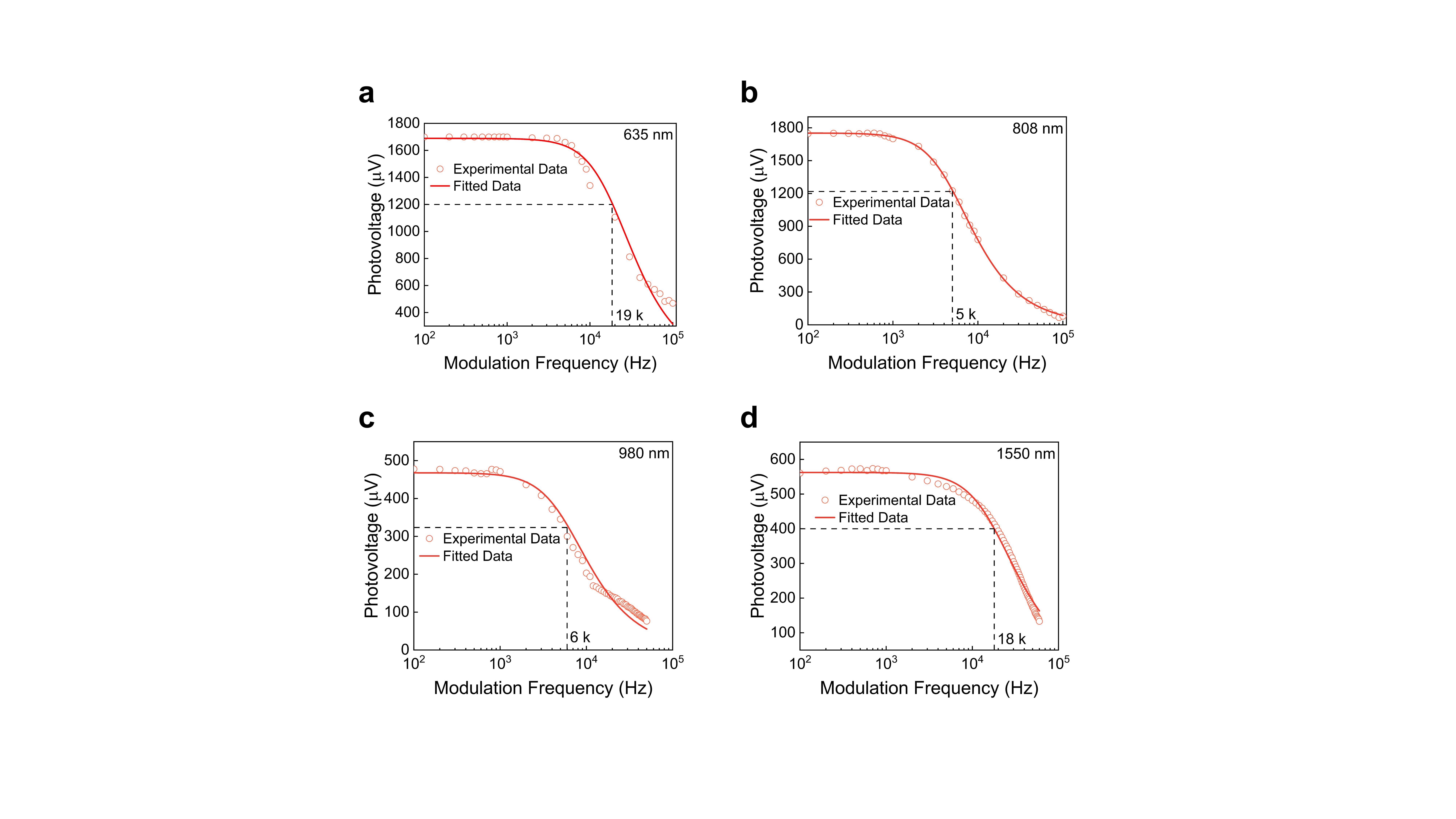
**

Fig.S10 The ambient electrical bandwidth at VIS, NIR and SWIR wavelengths. **a,** 635 nm. **b,** 808 nm. **c,** 980 nm. **d,** 1550 nm. The -3 dB frequency is measured as 19 kHz, 5 kHz, 6 kHz and 18 kHz, respectively.

**
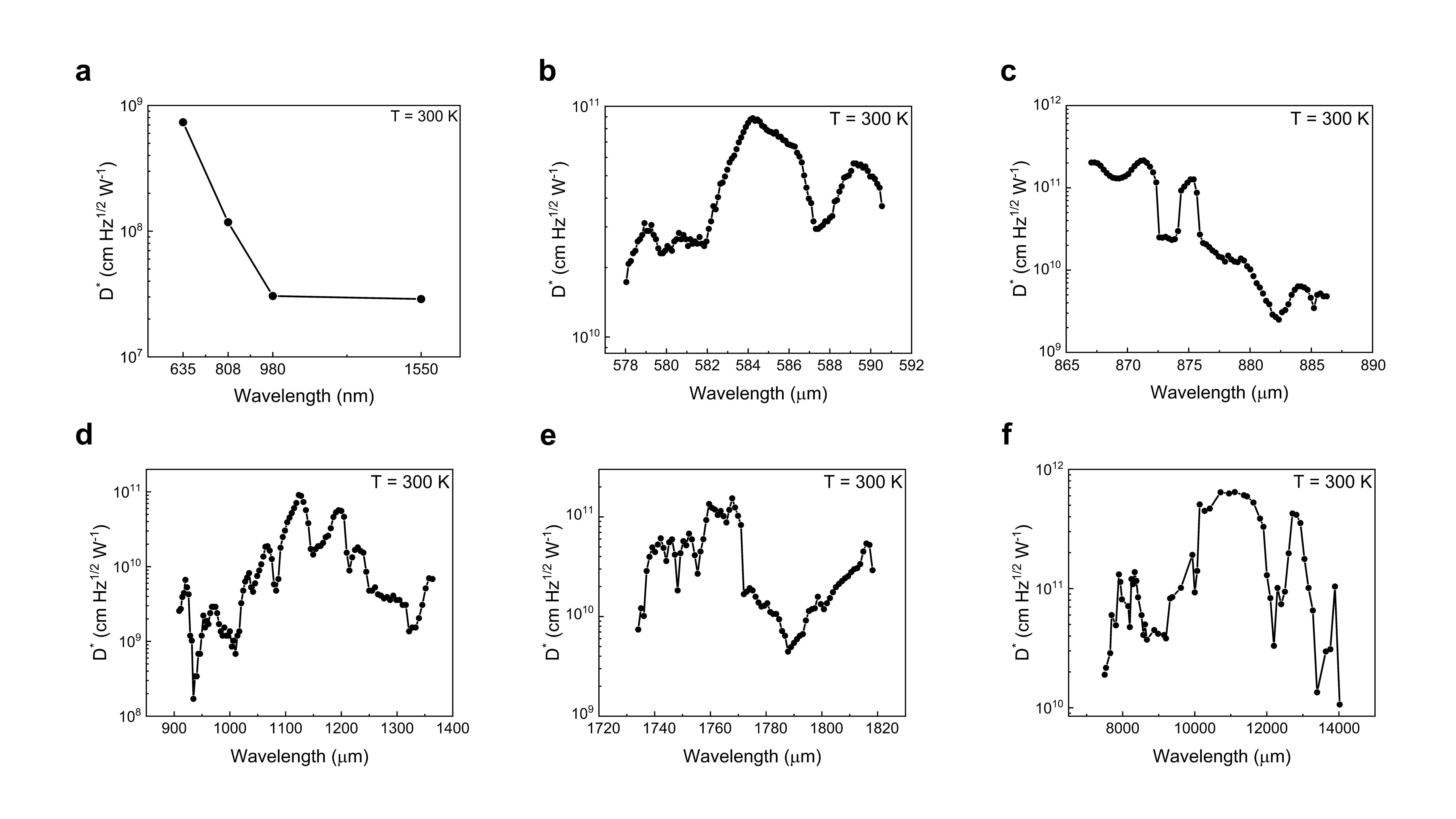
**

Fig.S11 The room-temperature D^*^ values of Ta_2_NiSe_5_ device at different wavelengths.

**
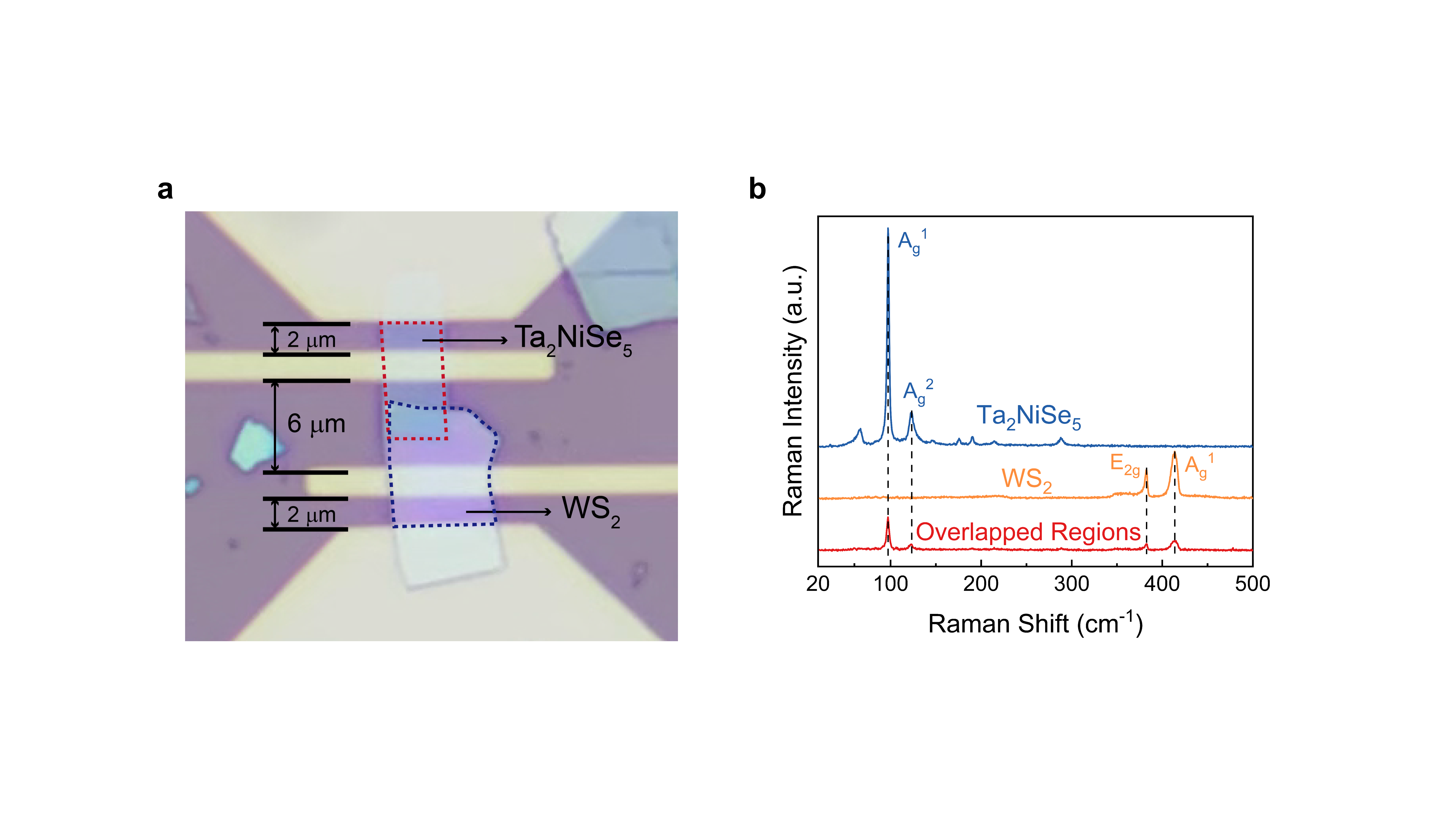
**

Fig.S12 The characterization of the Ta_2_NiSe_5_-WS_2_ vdW heterojunction. **a,** Optical microscopic images of the heterojunction. **b,** Raman characterization of the heterojunction.

**
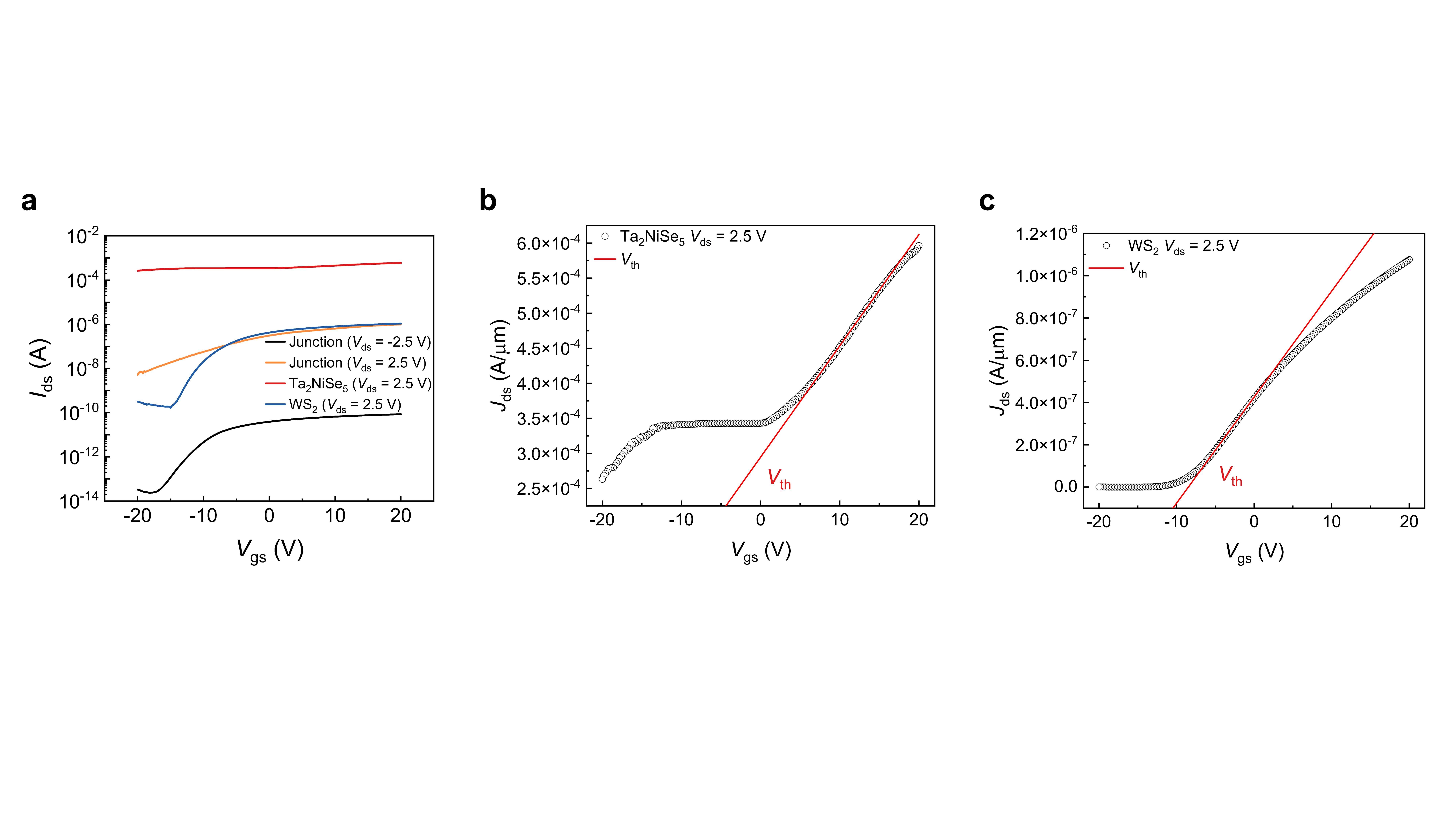
**

Fig.S13 Electrical characterization of the Ta_2_NiSe_5_-WS_2_ vdW heterojunction device. **a,** Transfer curves of individual devices in the four-terminal device. **b,** Mobility of Ta_2_NiSe_5_. $\mu=V_{th}\frac{L}{W}\frac{1}{C_{g}V_{ds}}$= 556.5 cm^2^ V^-1^ s^-1^, where *V*_th_ is the slope of the transfer curve, *L* is the channel length, *W* is the channel width, and *C*_g_ is the capacitance of the oxide layer. **c,** Mobility of WS_2_. $\mu=V_{th}\frac{L}{W}\frac{1}{C_{g}V_{ds}}$ = 1.8 cm^2^ V^-1^ s^-1^

**
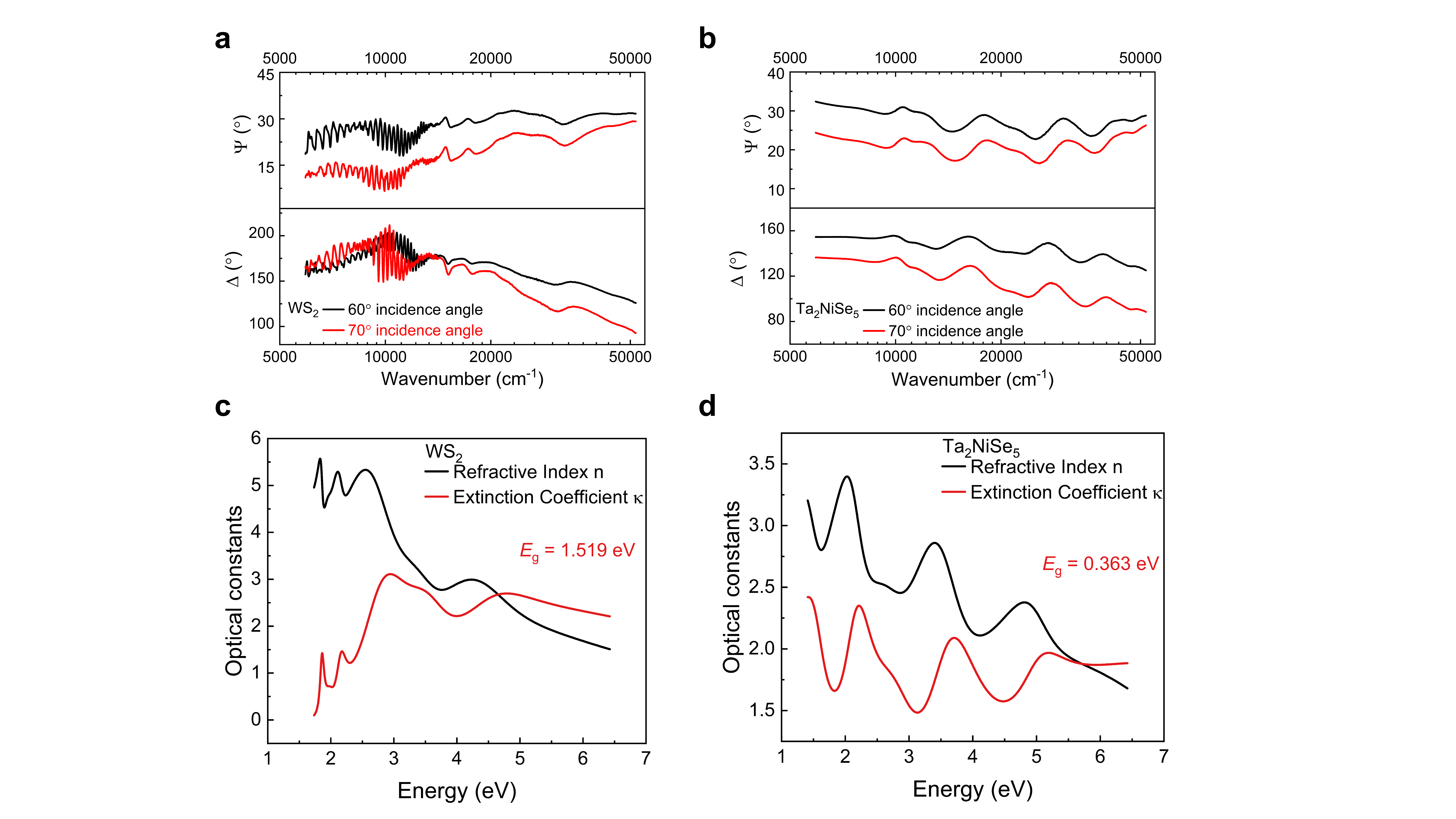
**

Fig.S14 Infrared spectroscopic ellipsometry (SE) characterization of Ta_2_NiSe_5_ and WS_2_. **a,** Relationship between the SE components Δ and Ψ of WS_2_ and the wave number of incident light. **b,** Relationship between the SE components Δ and Ψ of Ta_2_NiSe_5_ and the wave number of incident light. **c,** The refractive index (n) and extinction coefficient (κ) of WS_2_. The band gap of WS_2_ obtained through fitting is 1.519 eV. **d,** The refractive index (n) and extinction coefficient (κ) of Ta_2_NiSe_5_. The band gap of Ta_2_NiSe_5_ obtained through fitting is 0.363 eV. The optical permittivity of Ta_2_NiSe_5_ is described by a parameterized dispersive model^1,2^ consisting of five Tauc-Lorentz oscillators and two Gaussian oscillators, while the optical permittivity of WS_2_ is described by a model consisting of five Tauc-Lorentz oscillators and three Gaussian oscillators.

$\varepsilon_{2}\left( E \right)=\left\{ \begin{aligned} \sum_{i=1}^{5} \frac{A_{i}E_{0i}C_{i}\left( E-E_{gi} \right)^{2}}{\left( E^{2}-E_{0i}^{2} \right)^{2}+C_{i}^{2}E^{2}}\frac{1}{E} \left( E>E_{g} \right) \\ \\ 0 (E\leq E_{g}) \end{aligned} \right.$ (i = 1~5)

**
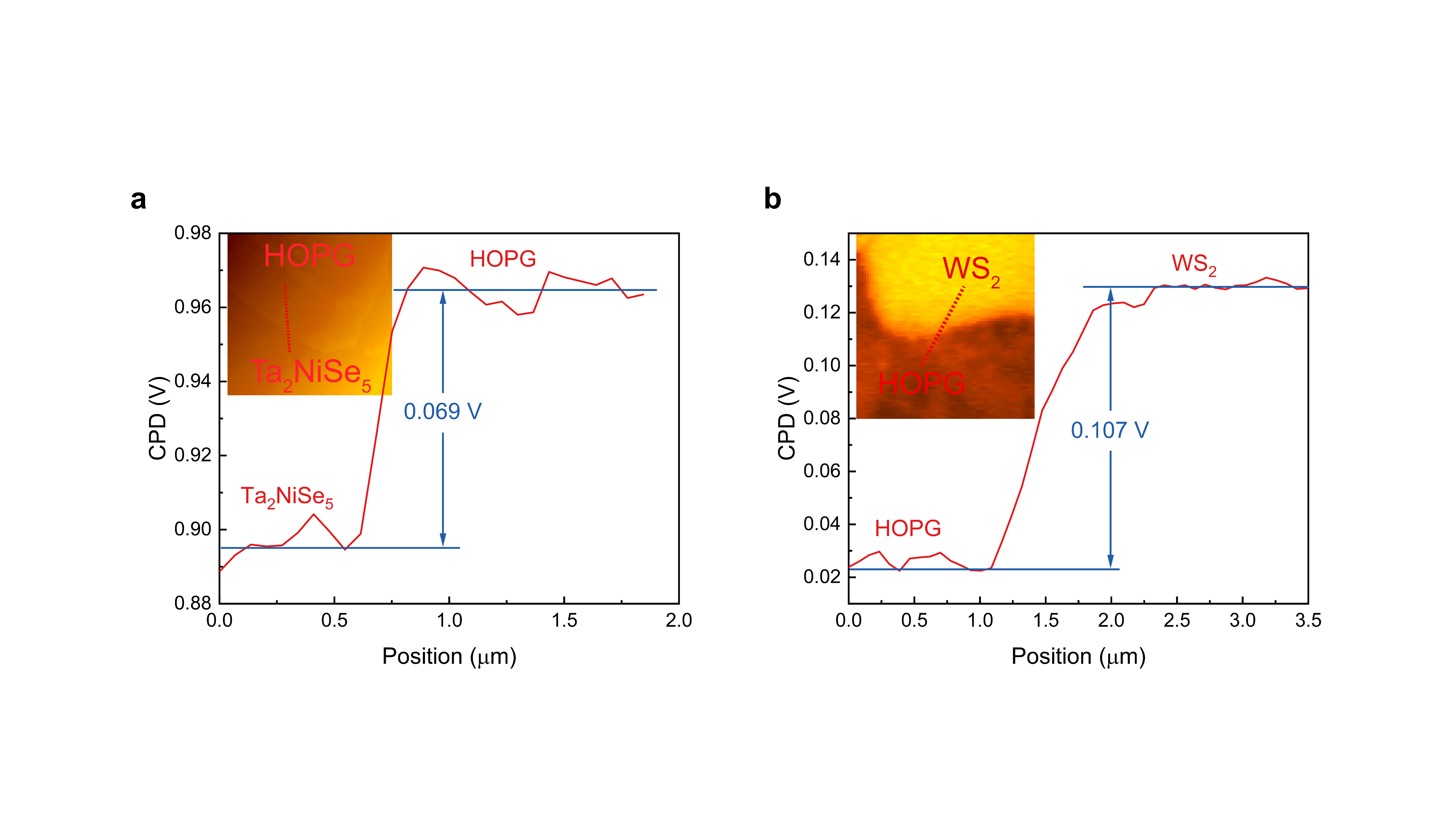
**

Fig.S15 Work function measurements of Ta_2_NiSe_5_ and WS_2_ by KPFM. **a,** Contact potential difference between Ta_2_NiSe_5_ and highly oriented pyrolytic graphite (HOPG) nanosheets. Inset: KPFM image of Ta_2_NiSe_5_ and HOPG nanosheets. **b,** Contact potential difference between WS_2_ and HOPG nanosheets. Inset: KPFM image of WS_2_ and HOPG nanosheets.

The HOPG is conventionally used to calibrate the work function for its clean surface and known work function of 4.6 eV. The potential difference between Ta_2_NiSe_5_ and HOPG is -0.069 V. The potential difference between WS_2_ and HOPG is 0.107 V. Therefore, the work function values of Ta_2_NiSe_5_ and WS_2_ can be estimated to be approximately 4.67 eV and 4.49 eV, respectively.

**
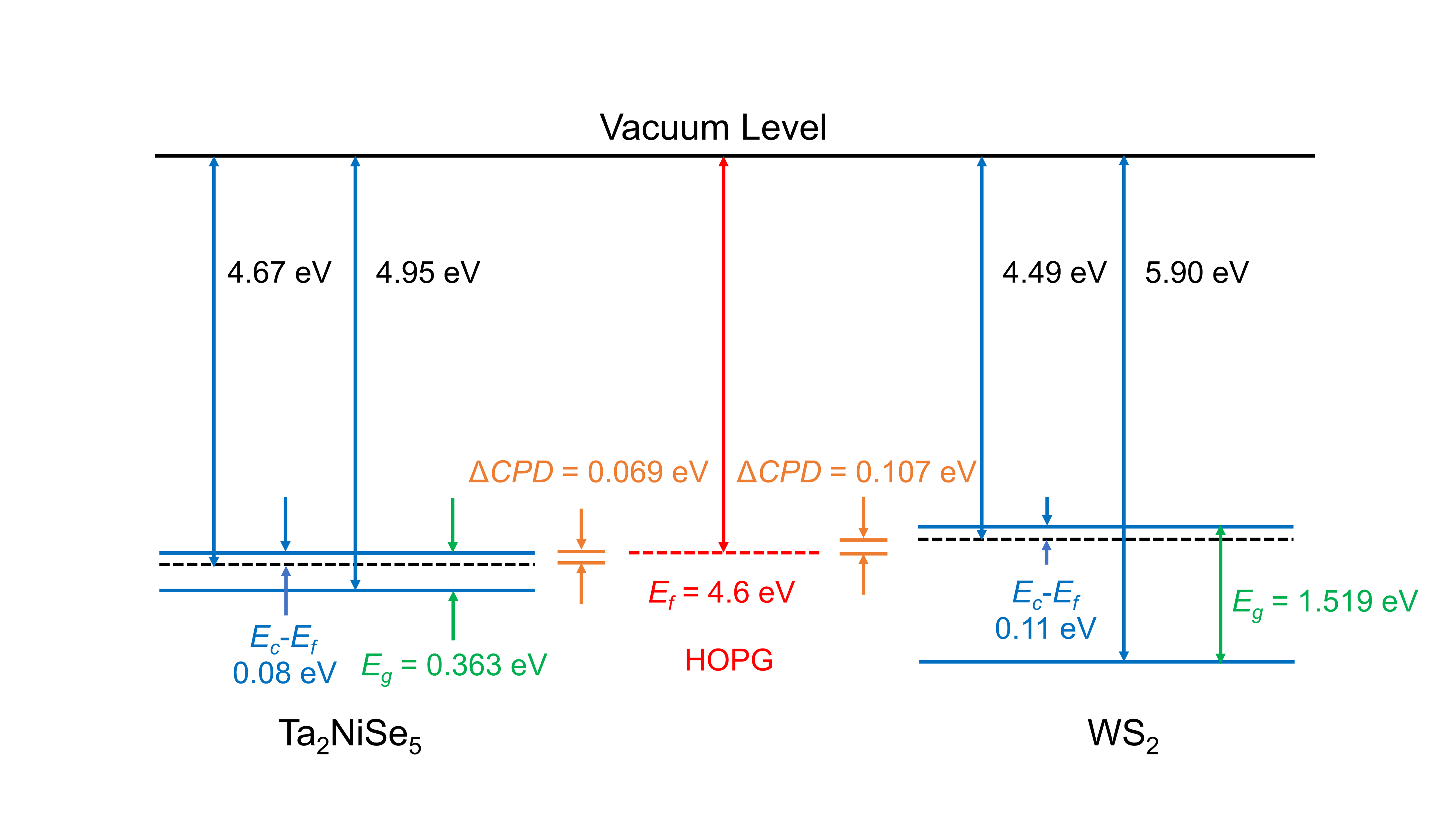
**

Fig.S16 Energy band alignments of Ta_2_NiSe_5_-WS_2_ vdW heterojunction by SE and KPFM. We calculate the difference between the Femi level and conduction band minimum as shown in Supplementary Note 2. For Ta_2_NiSe_5_ and WS_2_, we figure out (𝐸_𝐶_ − 𝐸_𝐹_) to be 0.08 eV for Ta_2_NiSe_5_ and 0.11 eV for WS_2_, respectively. In combination with the bandgap of Ta_2_NiSe_5_ and WS_2_, the valence band maximum of Ta_2_NiSe_5_ and WS_2_ are 4.95 eV and 5.90 eV, respectively.

**
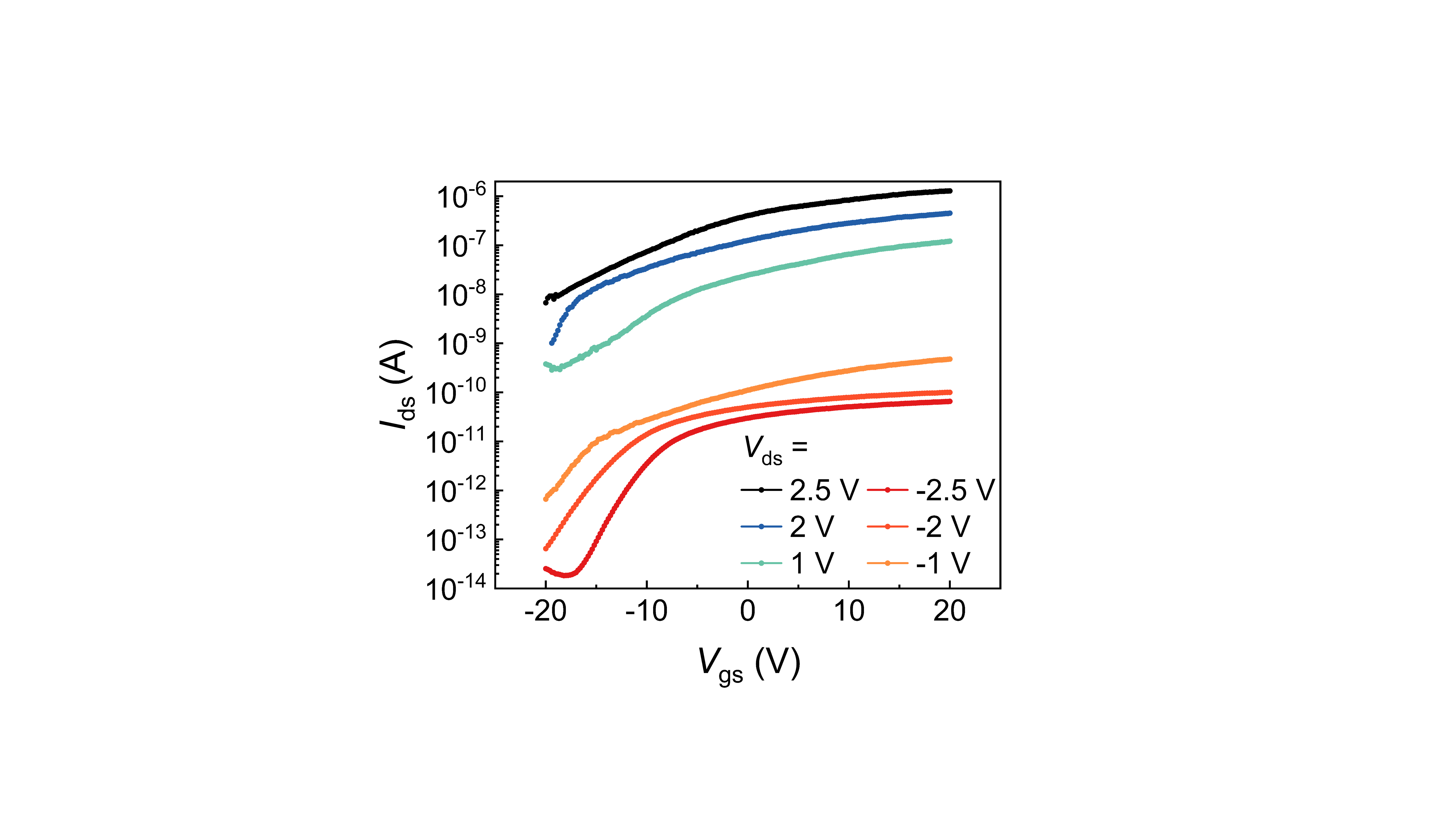
**

Fig.S17 Transfer curves of Ta_2_NiSe_5_-WS_2_ heterojunction device under different bias voltages.

**
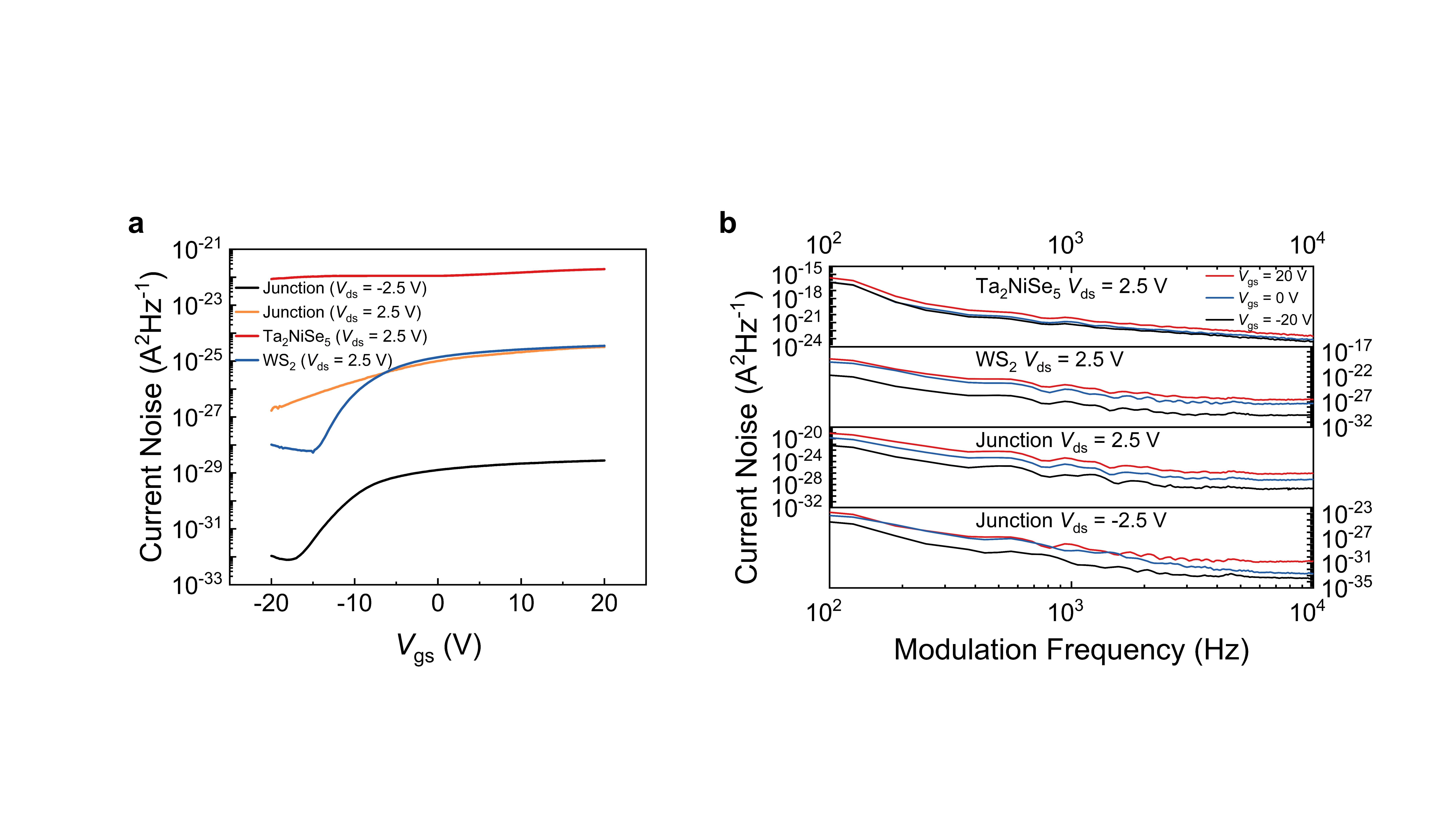
**

Fig.S18 Noise characterization of the four-terminal device. **a,** Variation of current noise in individual devices within the four-terminal device with respect to gate voltage. **b,** Comparison of current noise under different gate voltages for each device.

**
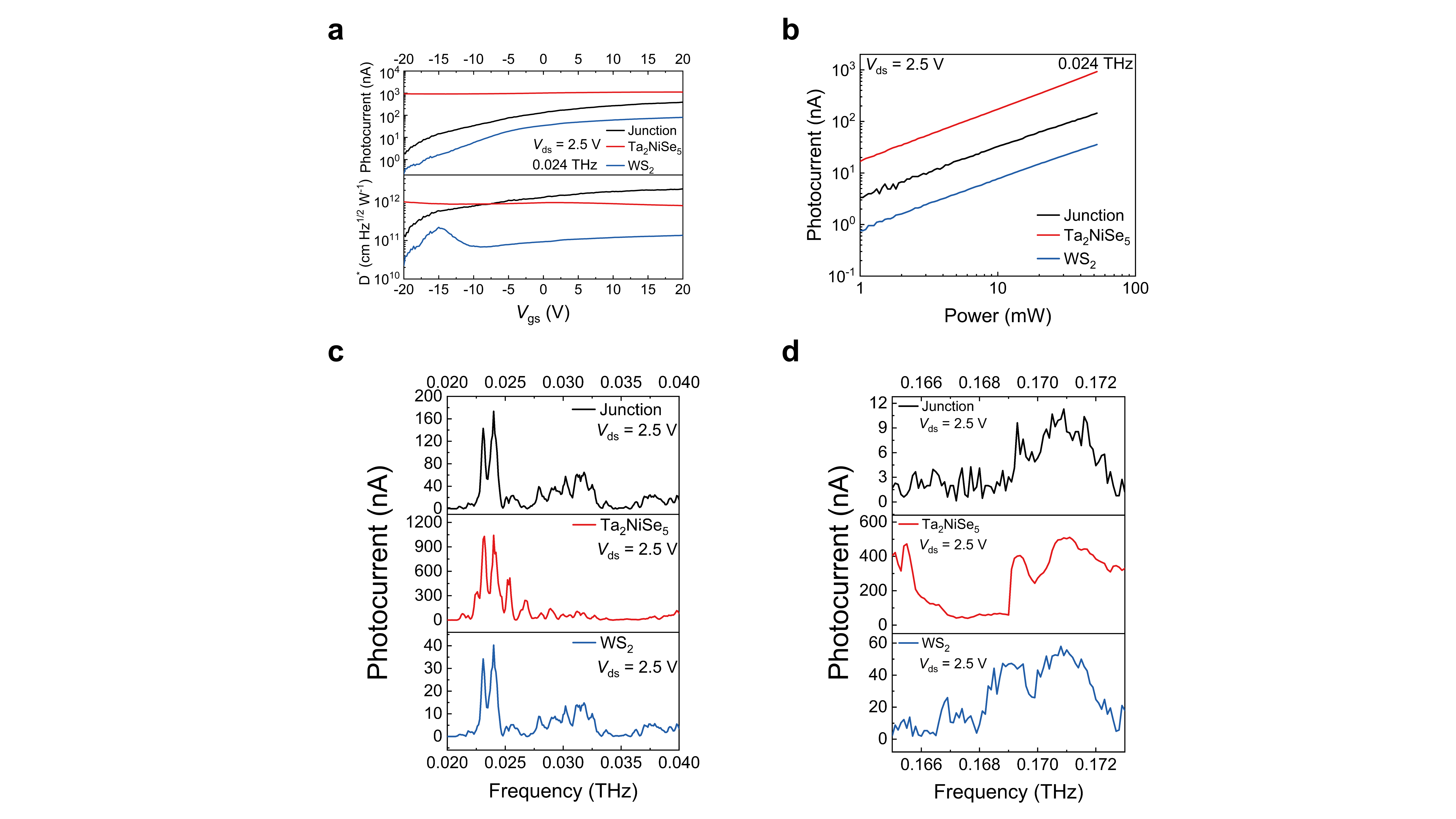
**

Fig.S19 Performance characterization of four-terminal device in the terahertz frequency range. **a,** Photocurrent and D^*^ of individual devices within the four-terminal device at 0.024 THz. **b,** Photocurrent of individual devices within the four-terminal device with increasing power. **c,** Response spectra of individual devices within the four-terminal device at 0.020-0.040 THz. **d,** Response spectra of individual devices within the four-terminal device at 0.163-0.173 THz.

**
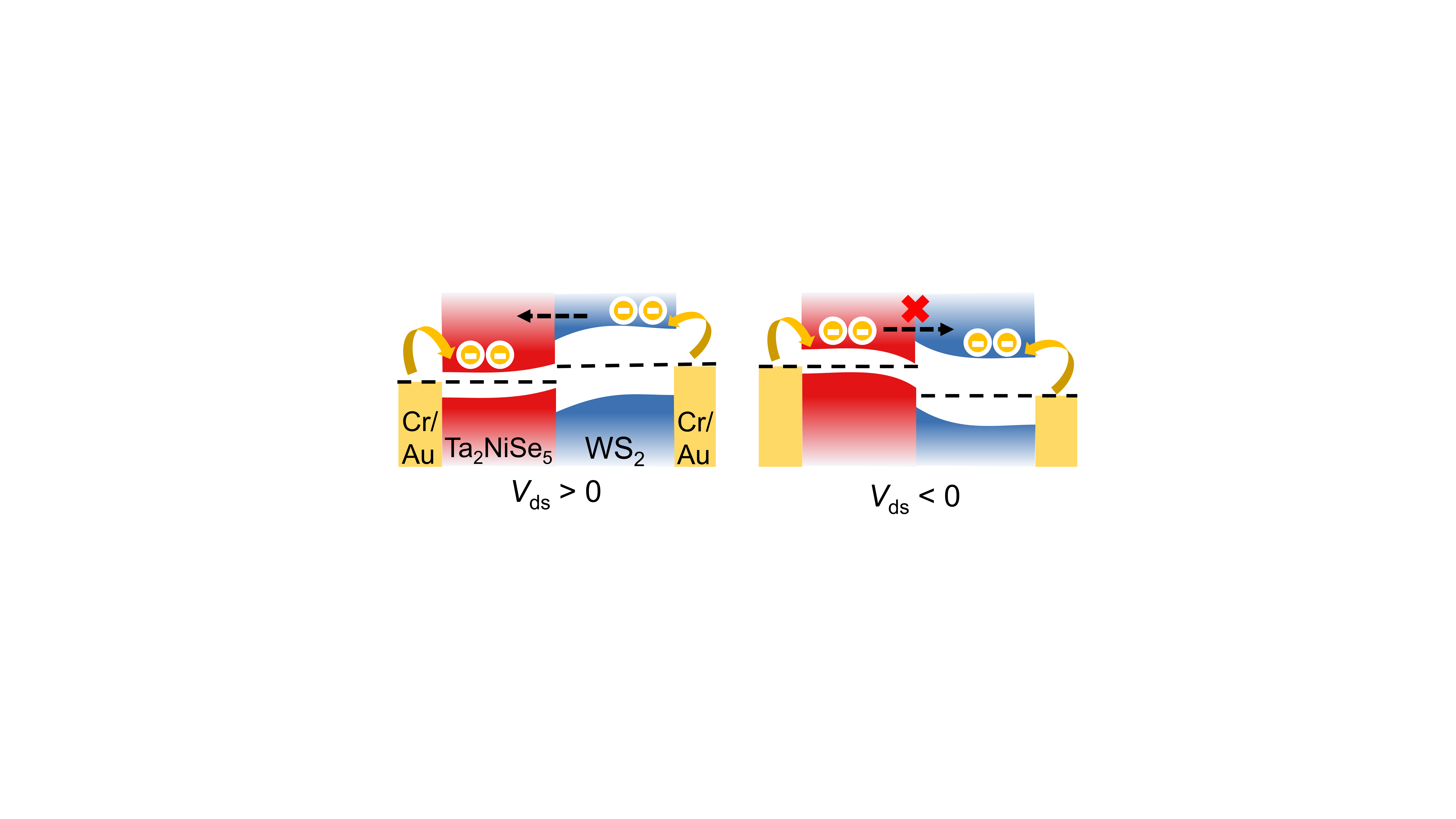
**

Fig.S20 Schematic illustration of the optoelectronic response of the Ta_2_NiSe_5_-WS_2_ vdW heterojunction device in the terahertz frequency range.

**
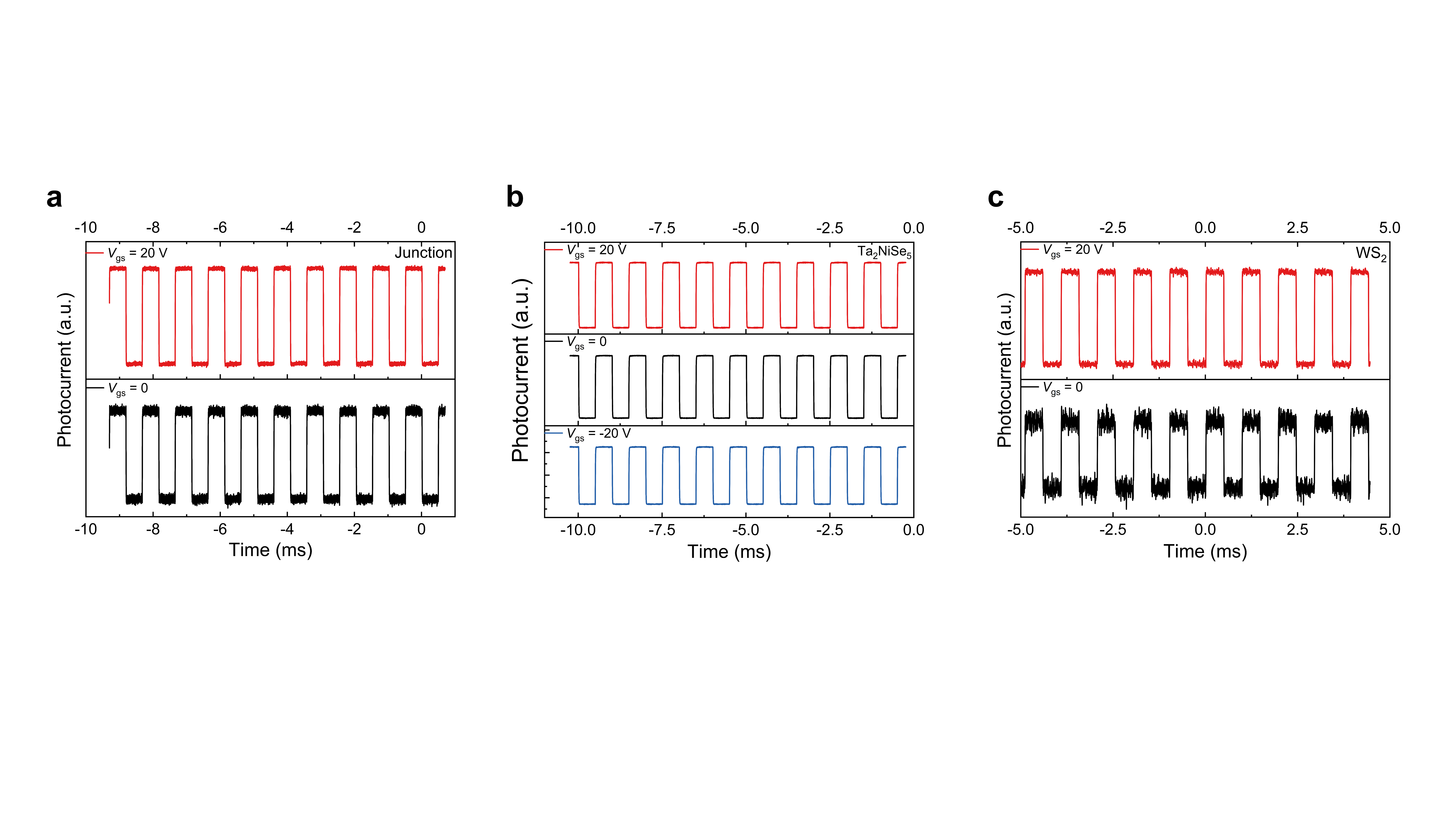
**

Fig.S21 Response waveforms of individual devices within the four-terminal device at 0.024 THz. **a,** Heterojunction device. **b,** Ta_2_NiSe_5_ device. **c,** WS_2_ device.

**
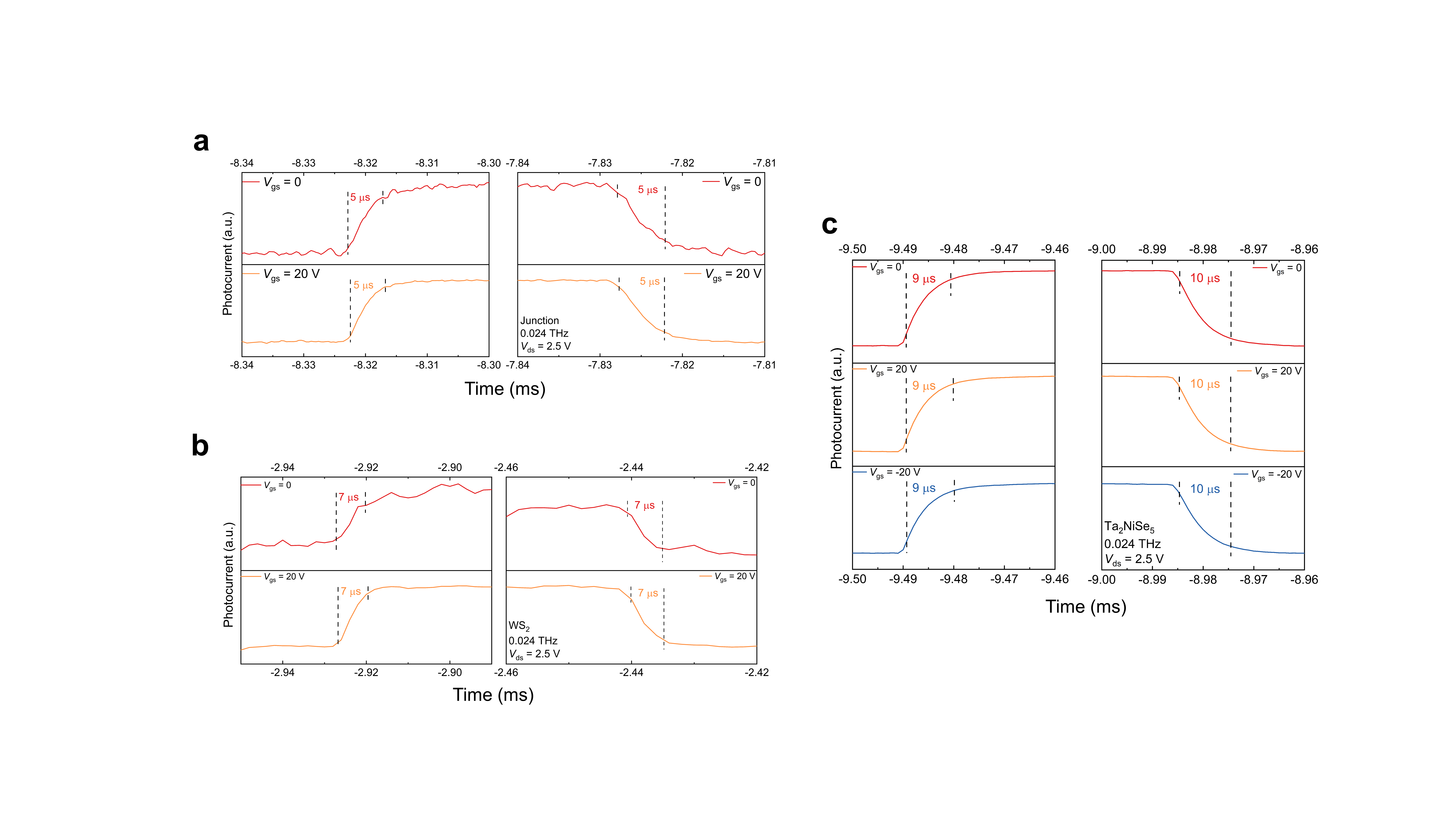
**

Fig.S22 Response time under variable gate voltage of individual devices within the four-terminal device at 0.024 THz. **a,** Heterojunction device. **b,** WS_2_ device. **c,** Ta_2_NiSe_5_ device.

**
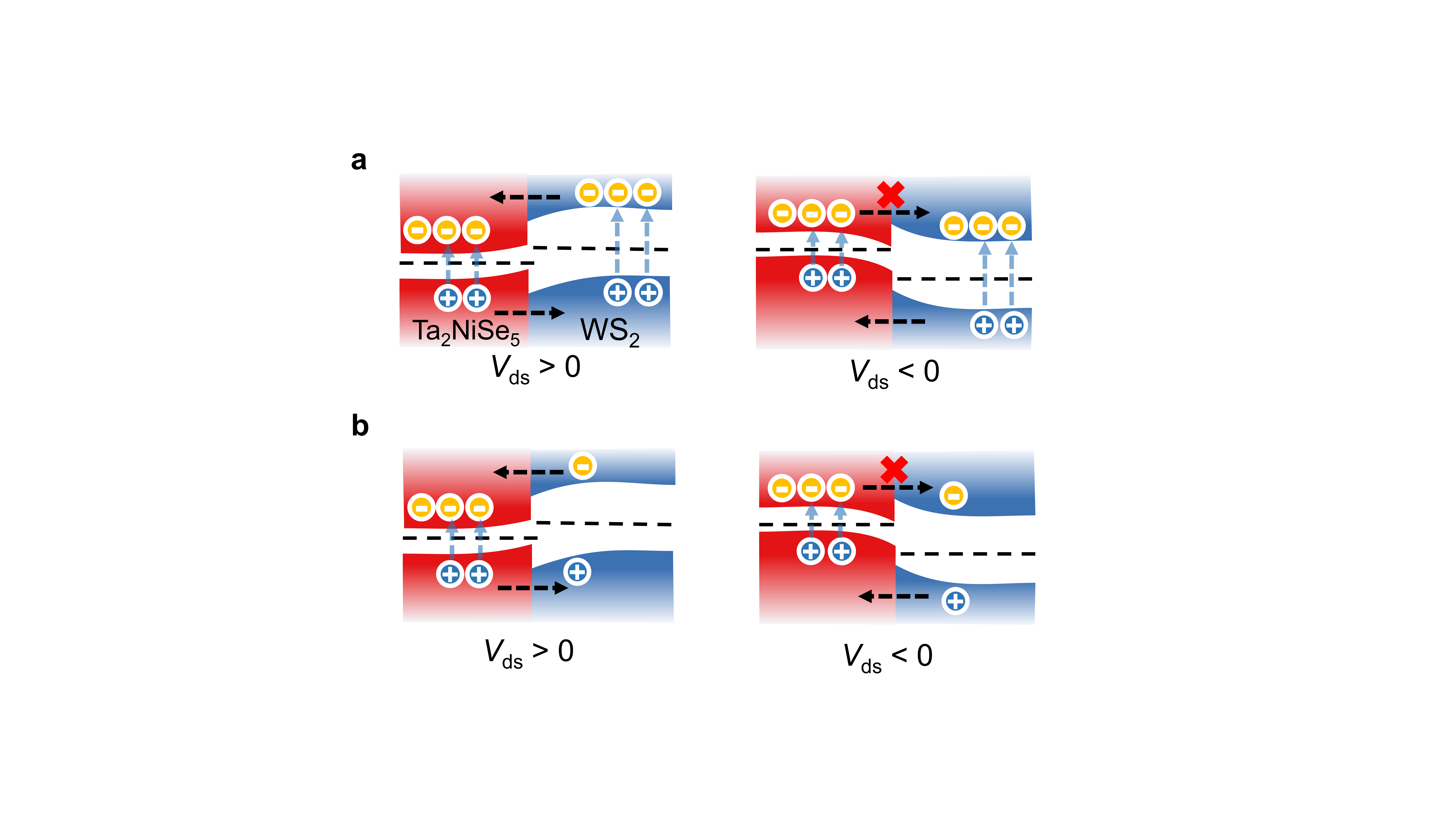
**

Fig.S23 Schematic illustration of the optoelectronic response of the Ta_2_NiSe_5_-WS_2_ vdW heterojunction device in the VIS, NIR and SWIR wavelength. **a,** 635 nm and 808 nm. **b,** 1550 nm.

**
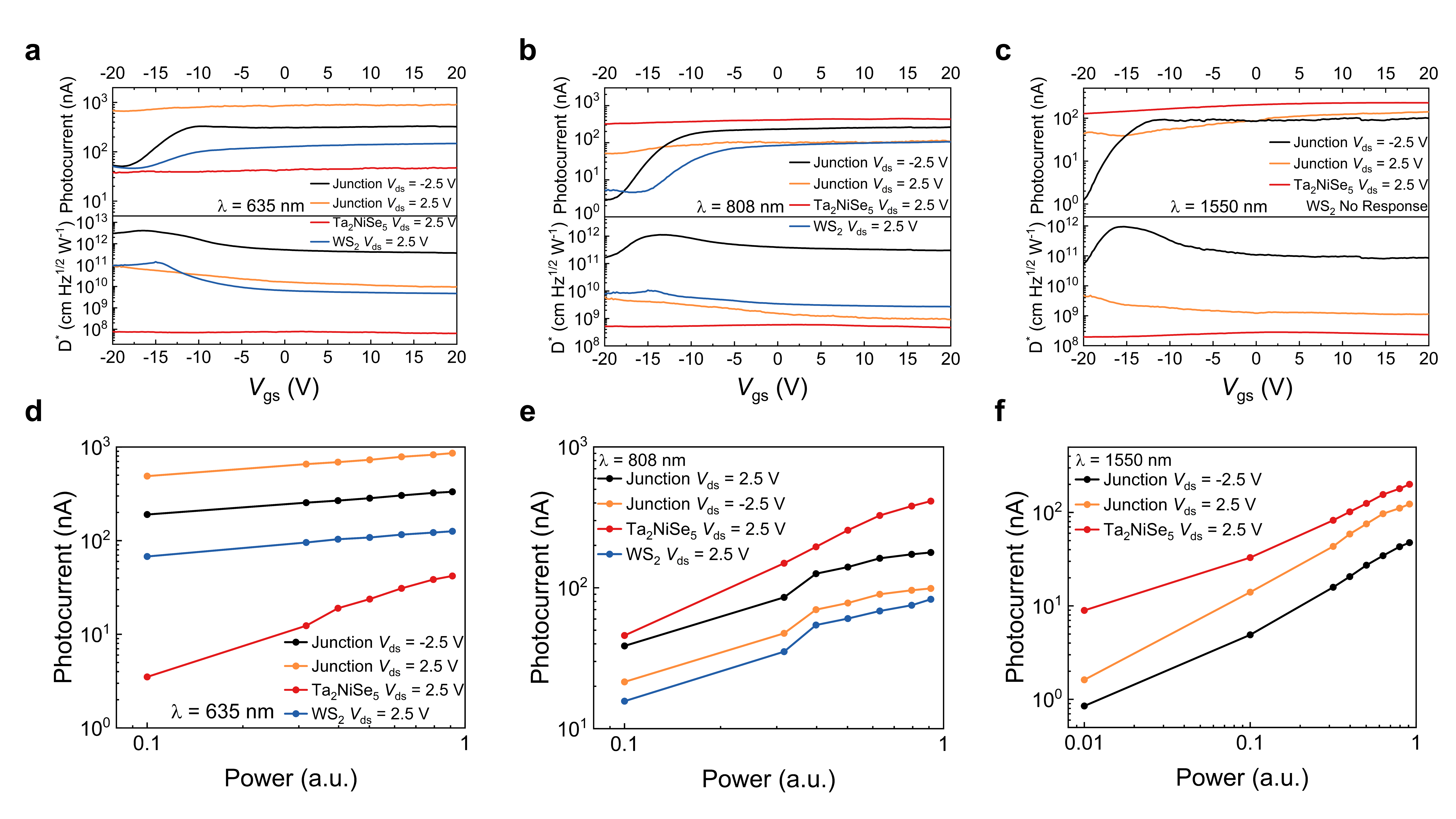
**

Fig.S24 Performance characterization of individual devices within the four-terminal device in the VIS, NIR and SWIR wavelength. **a,** Photocurrent and D^*^ at 635 nm for each device. **b,** Photocurrent and D^*^ at 808 nm for each device. **c,** Photocurrent and D^*^ at 1550 nm for each device. **d,** Photocurrent at 635 nm for each device with increasing power. **e,** Photocurrent at 808 nm for each device with increasing power. **f,** Photocurrent at 1550 nm for each device with increasing power.

**
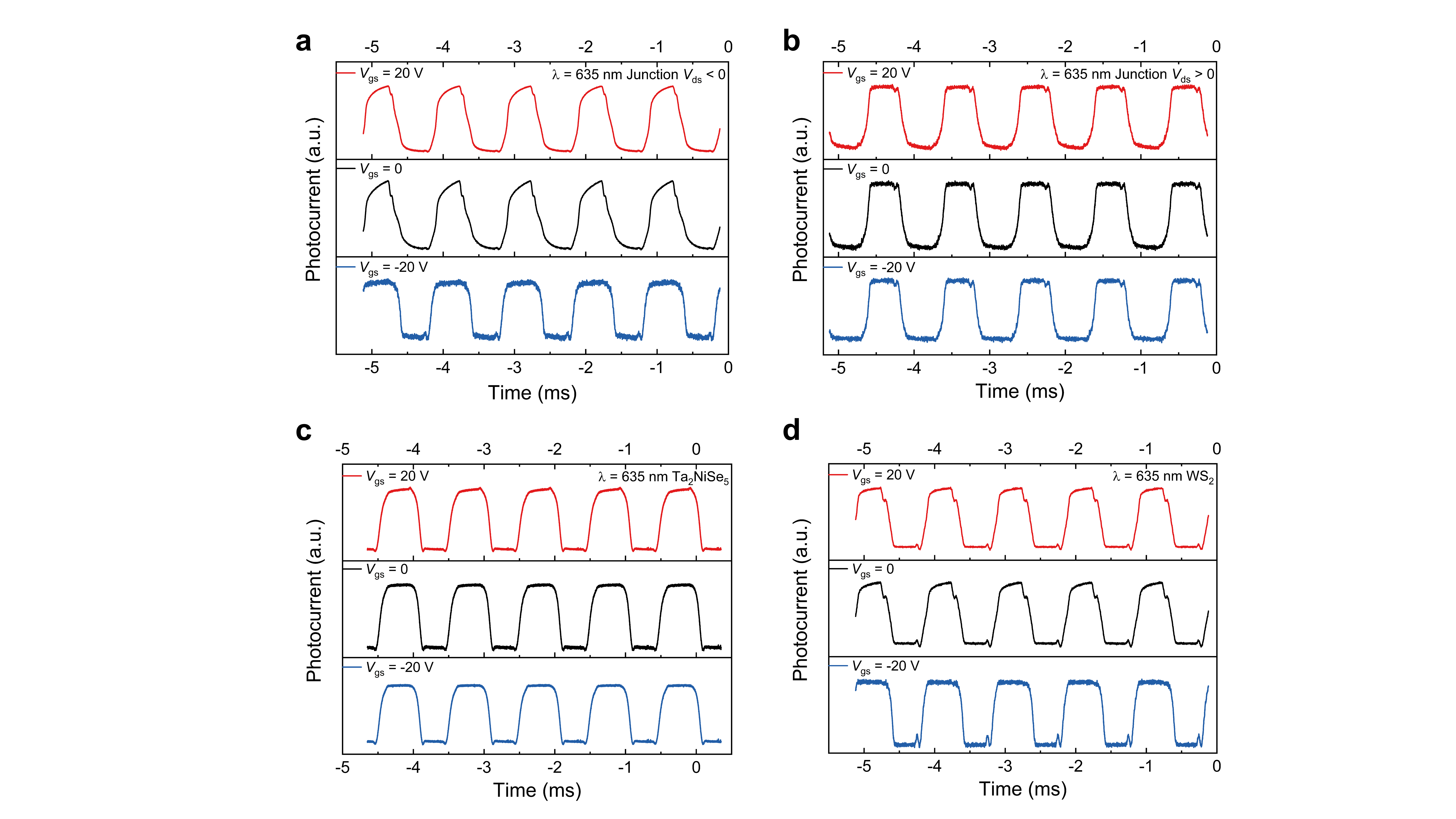
**

Fig.S25 Response waveforms of individual devices within the four-terminal device at 635 nm. **a,** Heterojunction device under reverse bias. **b,** Heterojunction device under forward bias. **c,** Ta_2_NiSe_5_ device. **d,** WS_2_ device.

**
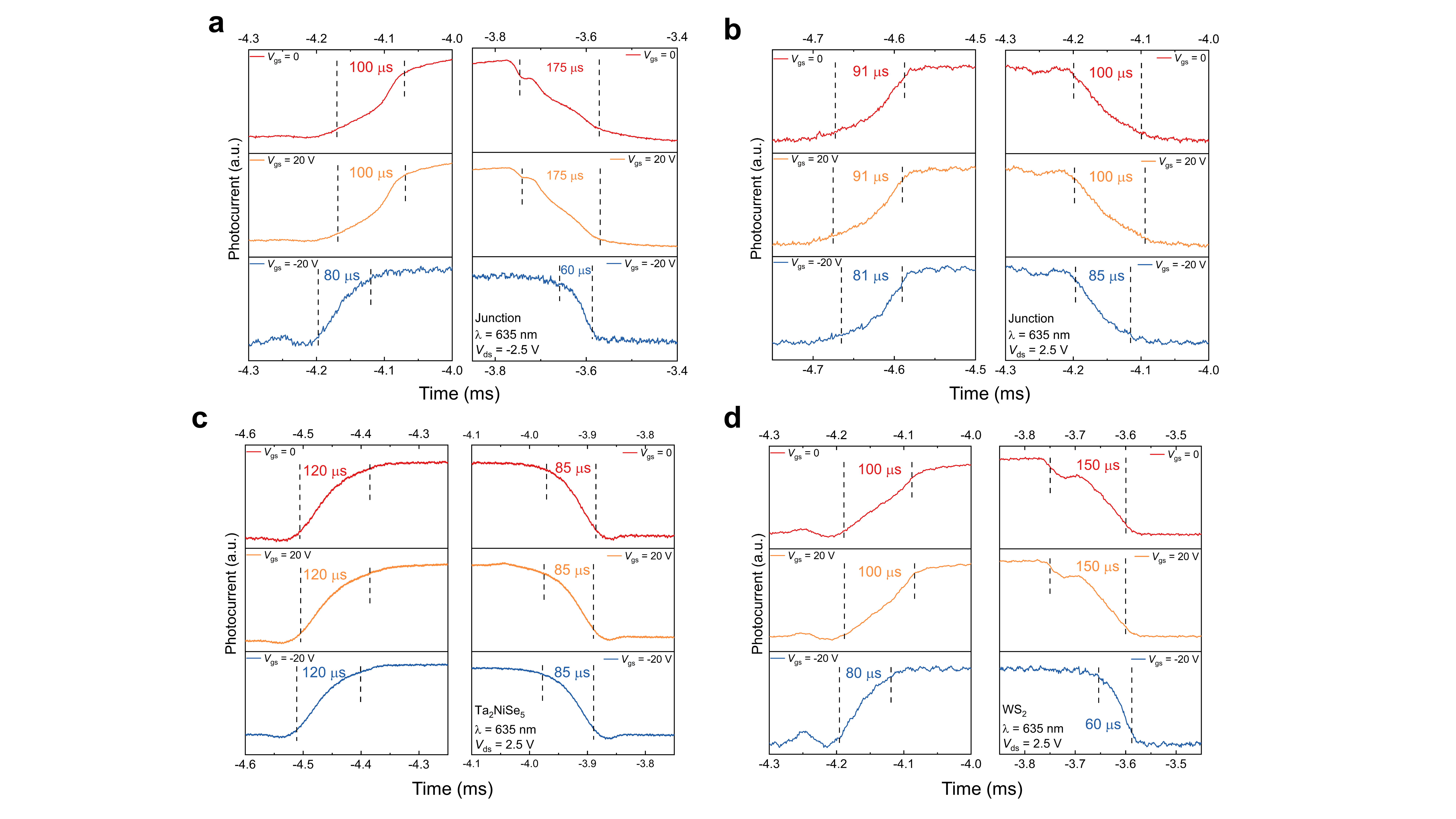
**

Fig.S26 Response time under variable gate voltage of individual devices within the four-terminal device at 635 nm. **a,** Heterojunction device under reverse bias. **b,** Heterojunction device under forward bias. **c,** Ta_2_NiSe_5_ device. **d,** WS_2_ device.

**
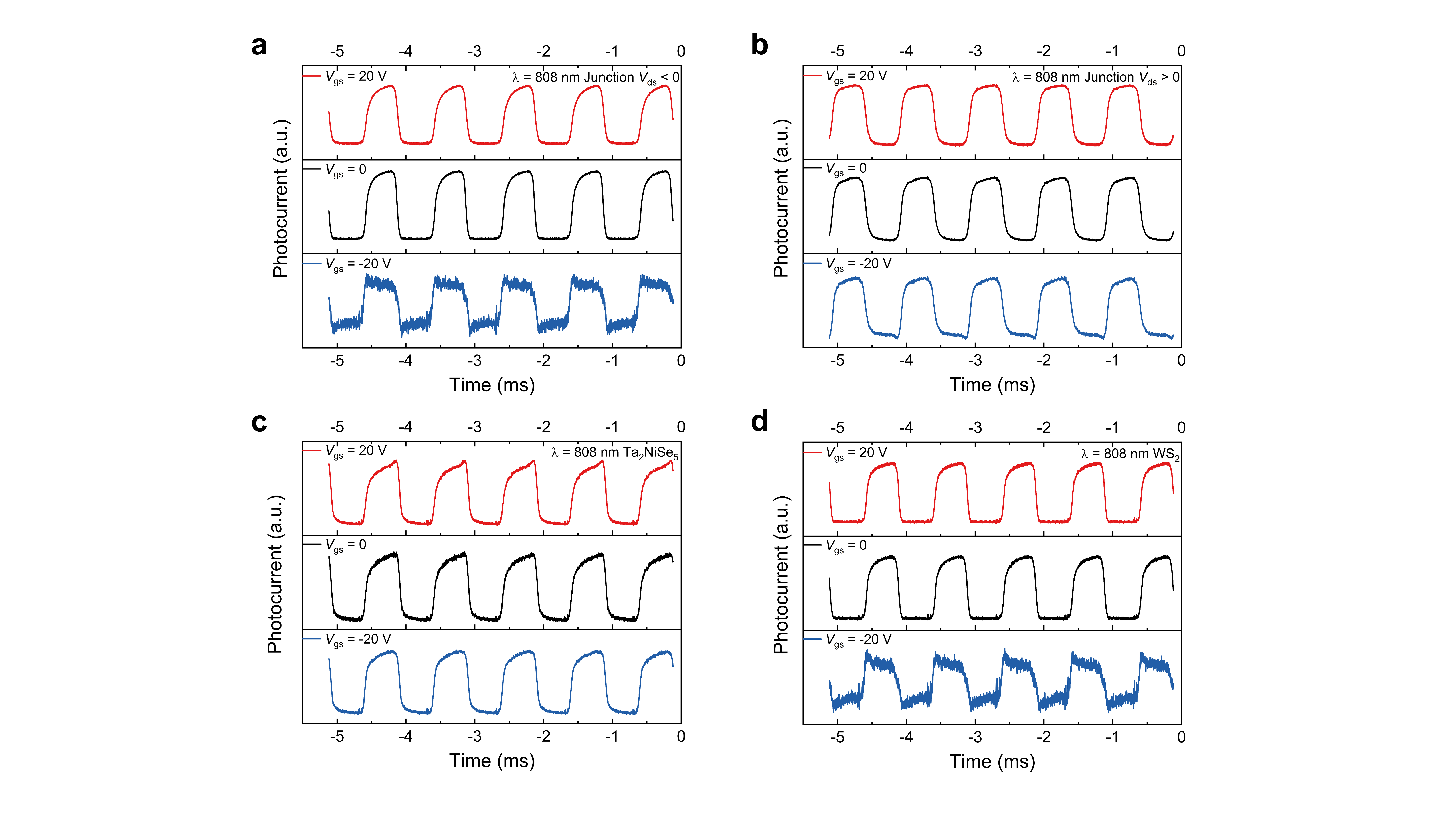
**

Fig.S27 Response waveforms of individual devices within the four-terminal device at 808 nm. **a,** Heterojunction device under reverse bias. **b,** Heterojunction device under forward bias. **c,** Ta_2_NiSe_5_ device. **d,** WS_2_ device.

**
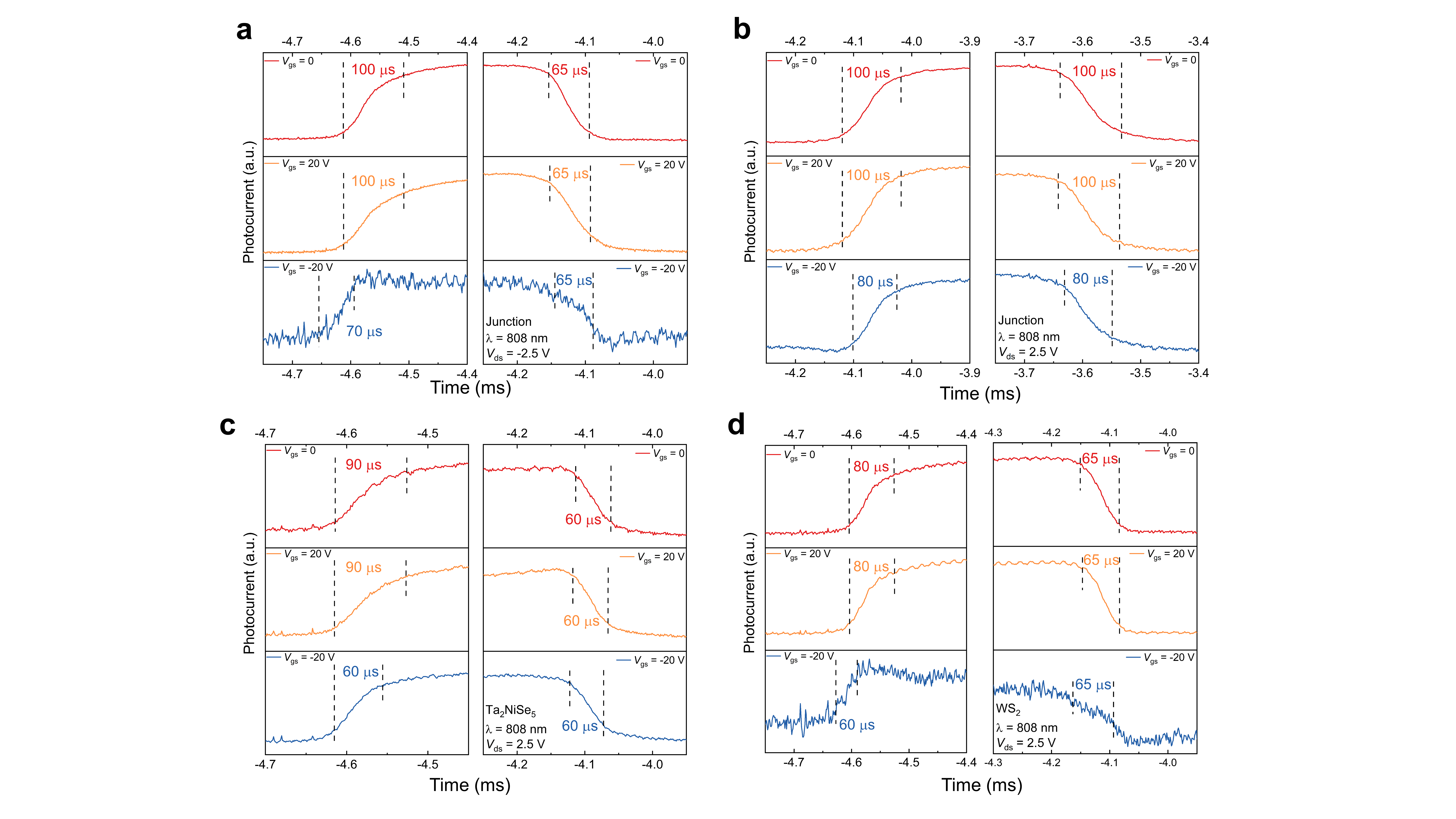
**

Fig.S28 Response time under variable gate voltage of individual devices within the four-terminal device at 808 nm. **a,** Heterojunction device under reverse bias. **b,** Heterojunction device under forward bias. **c,** Ta_2_NiSe_5_ device. **d,** WS_2_ device.

**
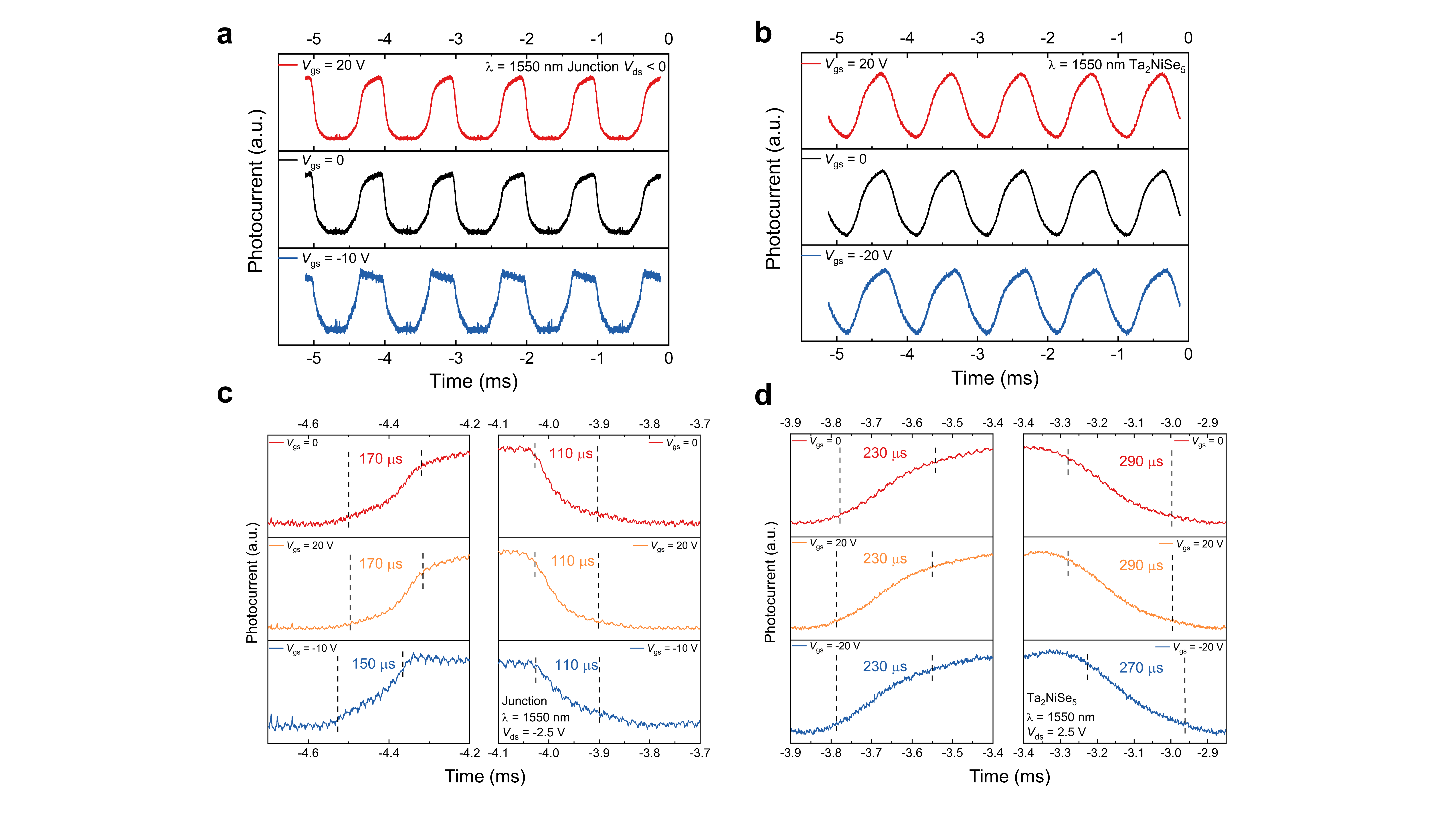
**

Fig.S29 Response waveforms and response time under variable gate voltage of individual devices within the four-terminal device at 1550 nm. **a,** Response waveform of heterojunction device under reverse bias. **b,** Response waveform of Ta_2_NiSe_5_ device. **c,** Response time of heterojunction device under reverse bias. **d,** Response time of Ta_2_NiSe_5_ device.

**
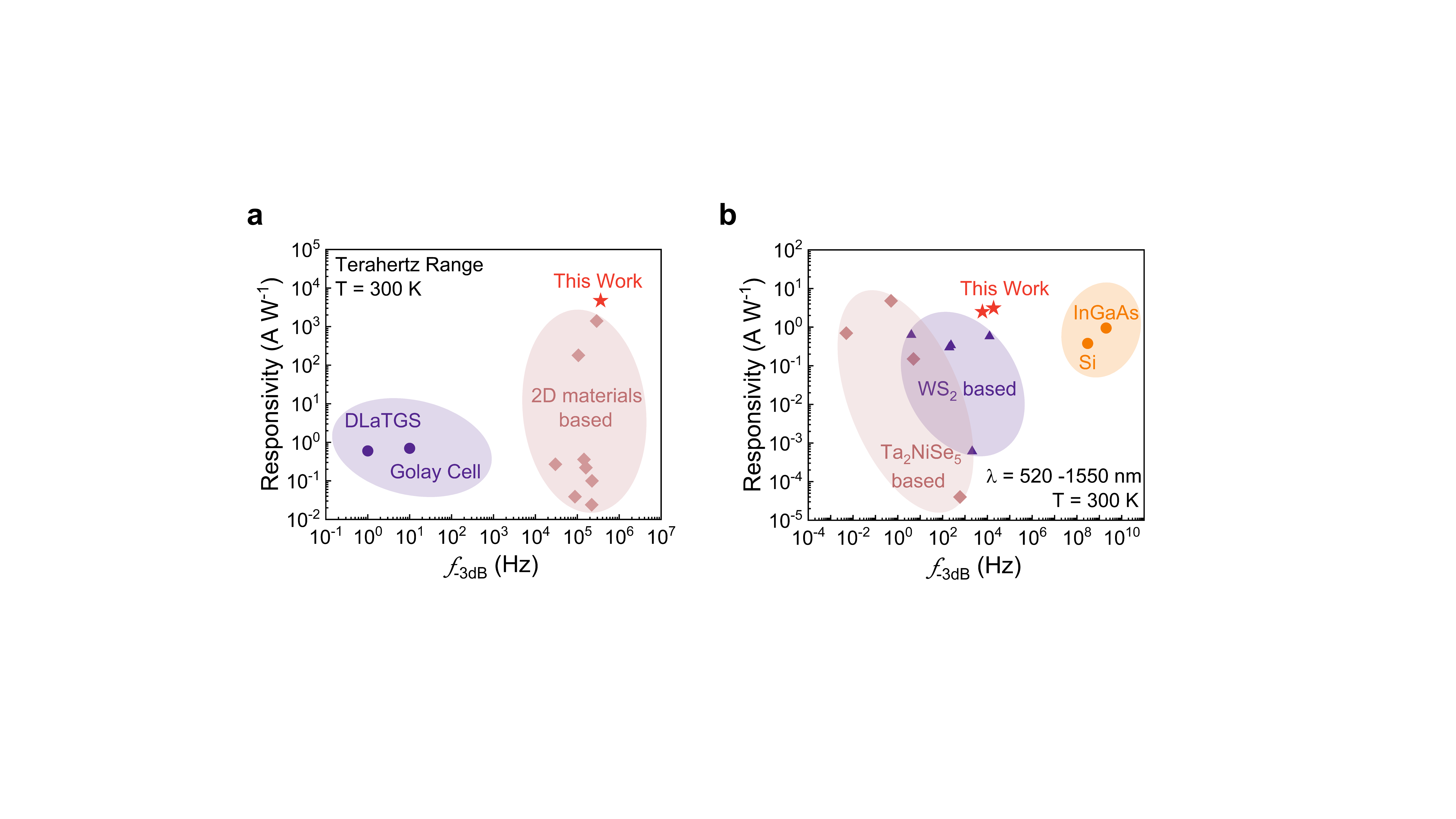
**

Fig.S30 Performance comparison of our photodetectors with previous photodetectors at room temperature. **a,** Responsivity and electrical bandwidth comparison of our photodetectors with commercial photodetectors and reported 2D material photodetectors in terahertz range. Detailed information can be found in Table S1. **b,** Responsivity and electrical bandwidth comparison of our photodetectors with commercial photodetectors and reported 2D material photodetectors at VIS, NIR and SWIR wavelengths. Detailed information can be found in Table S2.

For those references that did not explicitly state the electrical bandwidth of photodetectors, the *f*_-3dB_ is estimated using the equation $f_{-3dB}=\frac{1}{2\pi\tau_{rising}}$.

Table S1. Comparison on photoelectric performances in terahertz regime among reported 2D materials photodetectors and commercial devices at room temperature.

| Materials | Frequency [THz] | Response Time | Responsivity  [A∙W^-1^] | Detectivity D^*^  [cm·Hz^1/2^·W^-1^] | V_ds_  [V] | Refs |
| --- | --- | --- | --- | --- | --- | --- |
| Ta_2_NiSe_5_ | 0.340-0.346  0.508-0.519 | 500 ns | 4.7×10^3^  1.9×10^3^ | 8.9×10^10^  2.2×10^11^ | 0.1 | This Work |
| Ta_2_NiSe_5_-WS_2_ | 0.165-0.173 | 5 μs | 4.5×10^1^ | 7.0×10^11^ | 2.5 | This Work |
| Ta_2_NiSe_5_-Graphene | 0.12  0.30 | 720 ns | 2.4×10^-2^  1.8×10^-2^ | 3.4×10^7^  2.5×10^7^ | 0.1 | [3] |
| PtSe_2_-Graphene | 0.30 | — | 2.0×10^-1^ | 1.3×10^9^ | 0 | [4] |
| PdSe_2_-Graphene | 0.12  0.30 | 5.4 μs | 2.7×10^-1^  7.2×10^-2^ | 1.3×10^8^  2.5×10^6^ | 0.1 | [5] |
| CoTe_2_ | 0.24 - 0.30 | 710 ns | 1.0×10^-1^ | 2.8×10^10^ | 0.1 | [6] |
| PdTe_2_ | 0.30 | 1 μs | 2.2×10^-1^ | 2.5×10^10^ | 0.1 | [7] |
| Graphene | 0.167 | 1.5 μs | 1×10^2^ | 9.3×10^8^ | 0.1 | [8] |
|  | 0.035 |  | 1.8×10^2^ | 1.7×10^9^ |  |  |
| Ta_2_NiSe_5_ | 0.255 - 0.285 | 550 ns | 1.4×10^3^ | 5.5×10^10^ | 0.1 | [9] |
| Ta_2_NiSe_5_ | 0.1  0.3 | 1.1 μs  1.8 μs | 3.6×10^-1^  3.9×10^-2^ | 1.3×10^10^  7.5×10^8^ | 0.1 | [10] |
| Commercial Devices | | | | | | |
| Golay Cell | 0.04 - 750 | 30 ms | (1×10^4^ V∙W^-1^) | 7.0×10^9^ | 220 | [11] |
| DLaTGS | 0.15 - 500 | 140 ms | 6×10^-1^ | 1.8×10^9^ | 9 | [12,13] |
|  |  | 18 ms | 7×10^-1^ | 6.6×10^8^ | 9 |  |

Table S2. Comparison on photoelectric performances in infrared regime among reported 2D materials photodetectors and commercial devices at room temperature.

| Materials | Wavelength [nm] | Response Time | Responsivity  [A∙W^-1^] | Detectivity D^*^  [cm·Hz^1/2^·W^-1^] | V_ds_  [V] | Refs |
| --- | --- | --- | --- | --- | --- | --- |
| Ta_2_NiSe_5_-WS_2_ | 635 | 80 μs | 3.1 | 4.1×10^12^ | 2.5 | This work |
|  | 808 | 100 μs | 2.5 | 1.2×10^12^ | 2.5 | This work |
|  | 1550 | 150 μs | 7.0×10^-1^ | 9.4×10^11^ | 2.5 | This work |
| Ta_2_NiSe_5_-MoS_2_ | 1064 | 31 s | 7.0×10^-1^ | 2.4×10^9^ | 3 | [14] |
| Ta_2_NiSe_5_-GaSe | 520 | 340 ms | 4.8 | 1.1×10^9^ | 3 | [15] |
|  | 1550 | 32 ms | 1.5×10^-1^ | 3.3×10^7^ | 3 | [15] |
| Ta_2_NiSe_5_-WSe_2_ | 638 | 283 μs | 4.0×10^-5^ | 3.6×10^5^ | 0 | [16] |
|  | 1550 | — | 8.2×10^-10^ | 9×10^1^ | 0 | [16] |
| WS_2_-InSe | 520 | 76 μs | 6.1×10^-4^ | 2.5×10^11^ | 1 | [17] |
| WS_2_-WSe_2_ | 405 | 671 μs | 3.4×10^-1^ | 3.3×10^11^ | 0 | [18] |
| WS_2_-Bi_2_O_2_Se | 532 | 38 ms | 6.3×10^-1^ | 9.5×10^8^ | 5 | [19] |
| WS_2_-NiTe_2_ | 532 | 800 μs | 3.0×10^-1^ | 2.4×10^9^ | 1 | [20] |
| WS_2_-Ge | 980 | — | 6.3×10^-1^ | 1×10^11^ | 0 | [21] |
|  | 1550 | 12.7 μs | 5.8×10^-1^ | 4.3×10^11^ | 0 |  |
| WS_2_-Si | 980 | — | 3.5×10^-1^ | 9×10^10^ | 0 | [21] |
|  | 1550 | — | 9.0×10^-2^ | 1.4×10^9^ | 0 |  |
| Ta_2_NiSe_5_ | 520 | — | 3.5×10^1^ | 3.5×10^10^ | 1 | [22] |
|  | 638 | — | 2.1×10^1^ | 3×10^10^ | 1 |  |
|  | 830 | — | 1.7×10^-1^ | 2×10^10^ | 1 |  |
|  | 1550 | 12 μs | 8.5 | 1.4×10^10^ | 1 |  |
| Ta_2_NiSe_5_ | 405 | 15.9 s | 1.4×10^2^ | 8.4×10^9^ | 1 | [23] |
|  | 785 | — | 2.6×10^1^ | 1.5×10^9^ | 1 |  |
|  | 1550 | — | 6.4×10^1^ | 3.8×10^9^ | 1 |  |
| Commercial Devices | | | | | | |
| Si | 190 - 1000 | 300 MHz (*f*_-3dB_) | 3.8×10^-1^ | 1.1×10^13^ | 5 | [24] |
| Si | 635  808  980 | —  —  — |  | 3.1×10^12^  3.6×10^12^  9.2×10^11^ | 5  5  5 | Characterized under the same experimental conditions |
| InGaAs | 900 - 1900 | 2 GHz  (*f*_-3dB_) | 9.5×10^-1^ | 1×10^12^ | 1 | [25] |

Note S1. Temperature-dependent photocurrent of Ta_2_NiSe_5_

Ⅰ. The variation in photocurrent of Ta_2_NiSe_5_ detectors above *T*_C_

Above *T*_C_, Ta_2_NiSe_5_ can be considered as a normal zero-gap semimetal. As is shown in Fig.S31, the carrier concentration decreases with decreasing temperature, while the mobility increases with decreasing temperature. For the terahertz frequency range, when low-energy terahertz photons are incident on the designed sub-wavelength MSM structure, the electrons from the metal electrodes will be injected and trapped in the well located at the semiconductor. The conductivity of the semiconductor will be consequently changed and photocurrent signal can be collected between the metallic contacts. The theoretical photovoltage is denoted as^26,27^:

$$V_{ph}=\frac{4\varepsilon_{0}\eta E_{0}V_{b}\tau\mu}{\pi^{3}q^{2}dc\sqrt{\varepsilon_{r}}n}\sqrt{\left( \frac{\pi}{a} \right)^{2}-k_{0}^{2}}\times\left[ 1-exp(-d\sqrt{\varepsilon_{r}}\sqrt{\left( \frac{\pi}{a} \right)^{2}-k_{0}^{2}} \right]$$

where *ε*_0_ is the permittivity in vacuum, *η* is the electric field enhancement factor, *E*_0_ is the electric field in free space, *V*_b_ is the bias voltage of the device, *τ* is the life time of the carriers, *q* is the unit electric charge, *ε*_r_ is the relative permittivity of semiconductor, *d* is the thickness of the detection material, *c* is the velocity of light in vacuum, *a* is the width of the gap, *k*_0_ is the wave vector of light in vacuum, *n* is the intrinsic carrier concentration and *μ* is the carrier mobility. Thus, it follows that $V_{ph}\propto\frac{\mu}{n}$. Consequently, in the terahertz frequency range, the photovoltage rises with decreasing temperature above *T*_C_.

Regarding the visible light and infrared wavelength, where the photon energy exceeds the bandgap energy, the electrons in the valence band or impurity bands will be excited to the conduction band, forming nonequilibrium electron–hole pairs. The electrons and holes can be collected by the electrodes under applied electric field, where the mobility of carriers play a vital role in photoelectric response. For the case where the conductivity is determined mainly by electrons, the conventional photoconductive responsivity can be expressed as^28^:

$$R_{v}=\frac{\eta_{0}\lambda V_{b}}{hclad}(\frac{1+b}{nb+p})\tau\cong\frac{\eta\lambda\tau V_{b}}{hcladn}$$

where *η*_0_ is the quantum efficiency, *λ* is the wavelength, *h* is the Plank constant, *l* is the length of the gap, *p* is the intrinsic hole concentration and *b* is the ratio of electron mobility to hole mobility. Thus, it follows that $V_{ph}\propto\frac{1}{n}$. Consequently, in the visible and infrared wavelength, the photovoltage rises with decreasing temperature above *T*_C_.


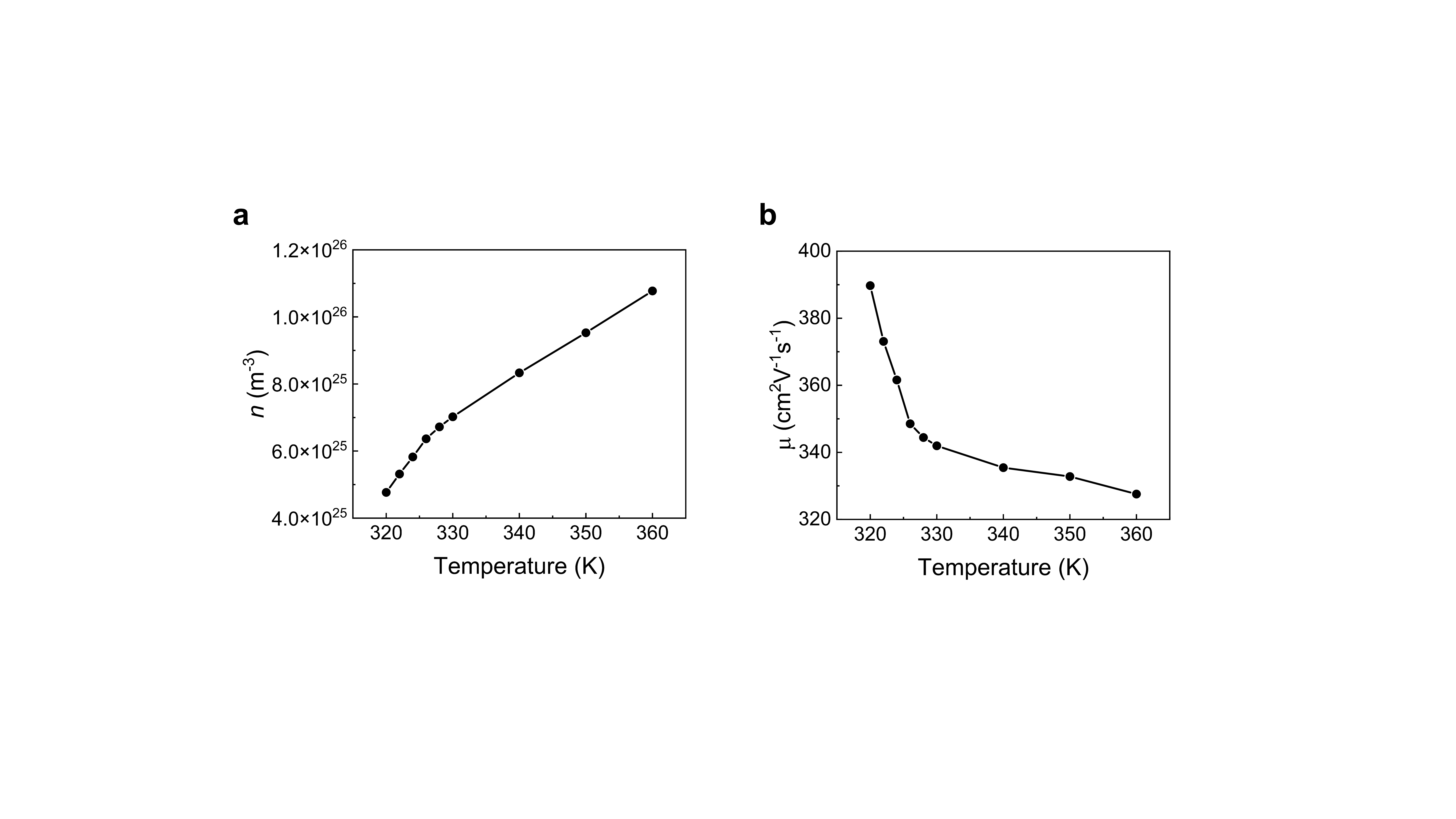


**Fig.S31 Electrical transport properties of Ta_2_NiSe_5_ above *T*_C_.**

Ⅱ. The variation in photocurrent of Ta_2_NiSe_5_ detectors below *T*_C_

Below *T*_C_, the emergence of the EI phase transition under the influence of BEC is observed in Ta_2_NiSe_5_, subsequently accompanied by a marked decrease in carrier concentration and a significant increase in mobility. The relative temperature coefficient of resistance (TCR) is defined as:

$$\alpha=\frac{1}{R}\frac{\partial R}{\partial T}=n\mu\frac{\partial\frac{1}{n\mu}}{\partial T}=-(\frac{1}{n}\frac{\partial n}{\partial T}+\frac{1}{\mu}\frac{\partial\mu}{\partial T})$$




**Fig.S32 The temperature coefficients of carrier concentration and mobility**

Thus, it can be inferred that the TCR is intimately linked to the carrier concentration and mobility temperature coefficients. Regarding to Fig.S32, the changing trends of both factors after the EI phase transition led to a sudden change in TCR below *T*_C_.

When the radiation input is received by the detector, the rise in temperature is found by solving the heat balance equation:

$$C_{th}\frac{\partial\Delta T}{\partial t}+G_{th}\Delta T=\varepsilon\Phi$$

where the detector is represented by a thermal capacitance *C*_th_ coupled via a thermal conductance *G*_th_ to a heat sink at a constant temperature, Δ*T* is the temperature difference due to optical signal Φ, and ε is the emissivity of detector.

Assuming the radiant power to be a periodic function,

$$\Phi=\Phi_{0}e^{i\omega t}$$

The solution of differential heat radiation is

$$\Delta T=\frac{\varepsilon\Phi_{0}R_{th}}{{(1+\omega^{2}\tau_{th}^{2})}^{1/2}}$$

where $\tau_{th}=C_{th}R_{th}$ is a characteristic thermal response time for the detect, and $R_{th}=1/G_{th}$ is the thermal resistance.

The relative temperature coefficient of conductivity can be deduced as

$$\frac{1}{\sigma}\frac{\partial\sigma}{\partial T}=\frac{1}{\sigma}\frac{l}{ad}\frac{\partial\frac{1}{R}}{\partial T}=-\frac{1}{R}\frac{\partial R}{\partial T}=-\alpha$$

Under terahertz radiation, the change of conductivity of a constant voltage-biased Ta_2_NiSe_5_ detectors below *T*_C_ is

$$\frac{\partial\sigma}{\partial T}=\frac{\partial(\sigma_{0}+\sigma_{EIW})}{\partial T}=-\alpha\sigma_{0}+n_{EIW}q\frac{\partial\mu}{\partial T}$$

where $n_{EIW}$ is the electrons injected by the EIW effect.

Similarly, under infrared laser illumination, the change of conductivity of a constant voltage-biased Ta_2_NiSe_5_ detectors below *T*_C_ is

$$\frac{\partial\sigma}{\partial T}=\frac{\partial(\sigma_{0}+\sigma_{PC})}{\partial T}=-\alpha\sigma_{0}+n_{PC}q\frac{\partial\mu}{\partial T}$$

where $n_{PC}$ is the electrons that transition from the valence band to the conduction band caused by the photoconductance effect.

The relative temperature coefficient of mobility can be defined as:

$$\beta=\frac{1}{\mu}\frac{\partial\mu}{\partial T}$$

The change of current of a constant voltage-biased Ta_2_NiSe_5_ detectors is

$$\Delta I=\frac{V_{0}}{R+\Delta R}-\frac{V_{0}}{R}=-\frac{{\Delta RV}_{0}}{R^{2}+R\Delta R}=-\frac{dl}{a}\frac{\frac{1}{\sigma_{0}}-\frac{1}{\sigma_{0}+\Delta\sigma}}{\frac{1}{{\sigma_{0}}^{2}}+\frac{1}{\sigma_{0}}(\frac{1}{\sigma_{0}}-\frac{1}{\sigma_{0}+\Delta\sigma})}V_{0}$$

$$=-\frac{dl}{a}\frac{\sigma_{0}\Delta\sigma}{\sigma_{0}+2\Delta\sigma}V_{0}\approx\frac{\alpha-\beta}{R}V_{0}\Delta T=\frac{(\alpha-\beta)V_{0}\varepsilon\Phi_{0}R_{th}}{{R(1+\omega^{2}\tau_{th}^{2})}^{1/2}}$$

Thus, we can conclude that for Ta_2_NiSe_5_ photodetector below *T*_C_, the photocurrent $I_{ph}$ is proportional to the $\frac{\alpha-\beta}{R}$ ($I_{ph}\propto\frac{\alpha-\beta}{R})$. This inference aligns with the results depicted in Fig.S33, where the photocurrent and (*α*-*β*)∙R^-1^ exhibit similar trends with temperature variation.


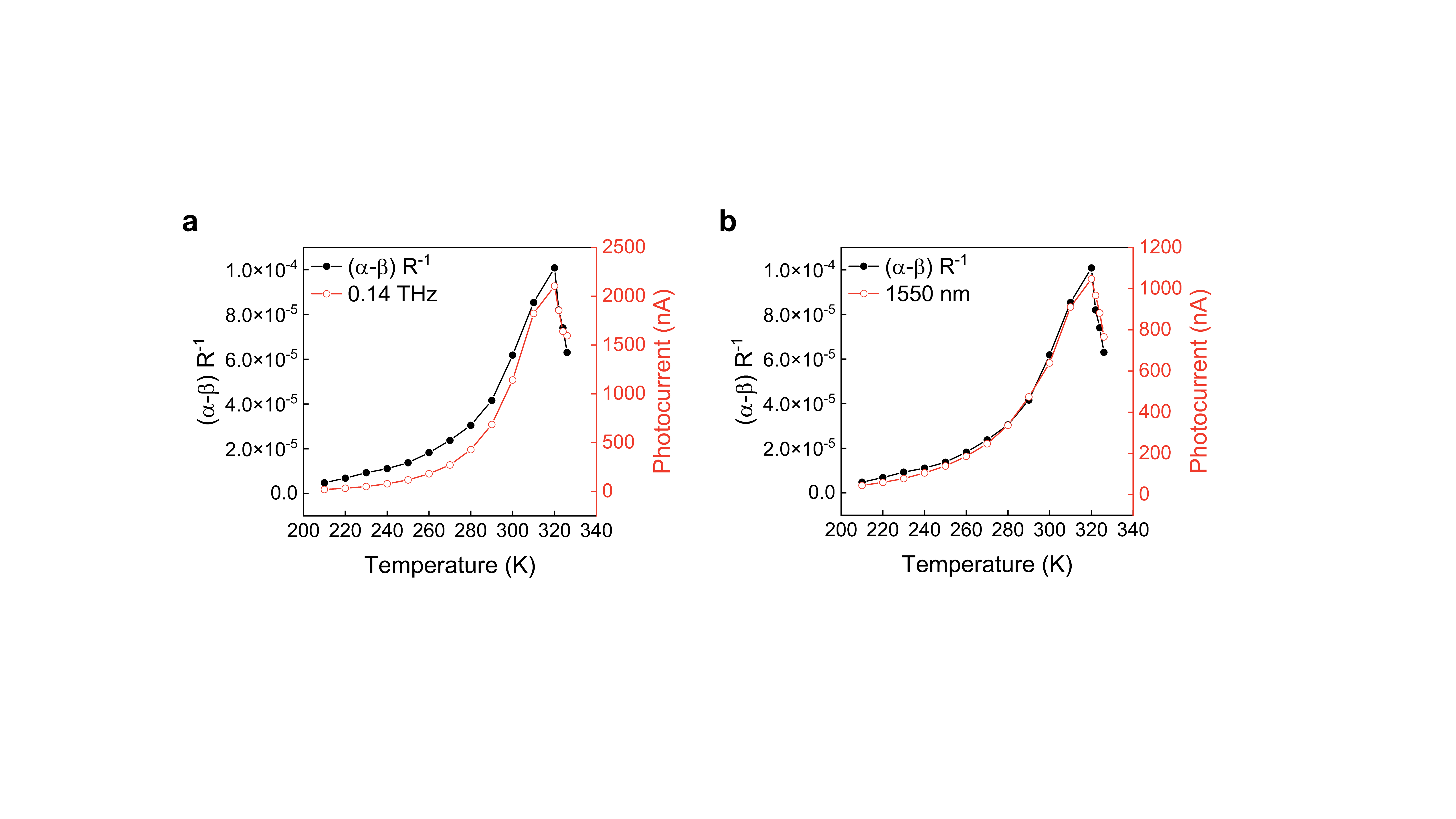


**Fig.S33 The variation trend of photoelectric response values in both terahertz ranges (a) and infrared wavelength (b) with temperature alongside theoretical predictions.**

Note S2. Band structure calculation of Ta_2_NiSe_5_ and WS_2_

We calculate the difference between the Fermi level and conduction band minimum according to the following formulas:

For nondegenerate n-type semiconductor (𝐸_𝐶_ − 𝐸_𝐹_ > 2𝑘_𝐵_𝑇)

$$N_{n}=N_{c}exp(-\frac{E_{c}-E_{F}}{k_{B}T})$$

For degenerate n-type semiconductor (𝐸_𝐶_ − 𝐸_𝐹_ ≤ 2𝑘_𝐵_𝑇)

$$N_{n}=N_{c}\frac{2}{\sqrt{\pi}}F_{1/2}(-\frac{E_{c}-E_{F}}{k_{B}T})$$

Where $N_{n}$ is the free electron volume concentration, $N_{C}=2\left( \frac{m_{e}^{*}k_{B}T}{2\pi\hbar^{2}} \right)^{3/2}$ is the conduction band equivalent density of states, (𝐸_𝐶_ − 𝐸_𝐹_) is the distance from Fermi level to conduction band minimum, 𝑘_𝐵_ is the Boltzmann constant, *T* is the temperature, $m_{e}^{*}$ is the electron effective mass, and $F_{1/2}$ is the Fermi integral function.

However, to obtain the position of Fermi level, the mobility and carrier concentration need to be calculated first. According to the measured electrical data, the mobilities of Ta_2_NiSe_5_ and WS_2_ are calculated to be 556.5 and 1.8 cm^2^ V^-1^ s^-1^. The carrier concentration can be calculated by $n=\frac{\delta}{\mu q}$, where $\delta$ is the conductivity in the linear region, $q$ is the electron charge, and $\mu$ is the mobility calculated above. In calculations, the volume of Ta_2_NiSe_5_ and WS_2_ were gained be $N_{n}=\frac{n}{h}$ to be 1.08×10^17^ cm^-3^ and 7.02×10^16^ cm^-3^, respectively, where $h$ is the thickness of analyzed 2D materials.$m_{e,{Ta}_{2}Ni{Se}_{5}}^{*}=0.23m_{0}$ and $m_{e,{WS}_{2}}^{*}=0.33m_{0}$ are adapt^29,30^. For lightly doped Ta_2_NiSe_5_ and WS_2_, we substituted $N_{n}$ and $N_{c}$ into formula (1) to figure out (𝐸_𝐶_ − 𝐸_𝐹_) to be 0.08 eV for Ta_2_NiSe_5_ and 0.11 eV for WS_2_.

Supplementary References

1 Fujiwara, H. & Kondo, M. Effects of carrier concentration on the dielectric function of ZnO:Ga and In_2_O_3_:Sn studied by spectroscopic ellipsometry: Analysis of free-carrier and band-edge absorption. *Phys. Rev. B* **71**, 075109 (2005).

2 Jellison, G. E. & Modine, F. A. Parameterization of the optical functions of amorphous materials in the interband region. *Appl. Phys. Lett.* **69**, 2137-2137, (1996).

3 Zhang, K. X. *et al.* Self-driven and ultrasensitive room-temperature terahertz photodetector based on graphene-Ta_2_NiSe_5_ van der Waals heterojunction. *Infrared Phys. Techn.* **128**, 104474 (2023).

4 Wang, L. *et al.* Hybrid Dirac semimetal-based photodetector with efficient low-energy photon harvesting. *Light:Sci. Appl.* **11**, 53 (2022).

5 Zhou, J. *et al.* A self-powered and sensitive terahertz photodetection based on PdSe_2_. *Chinese Phys. B* **31**, 050701 (2022).

6 Hu, Z. *et al.* Terahertz Nonlinear Hall Rectifiers Based on Spin-Polarized Topological Electronic States in 1T-CoTe_2_. *Adv. Mater.* **35**, 2209557 (2023).

7 Guo, C. *et al.* Anisotropic ultrasensitive PdTe_2_-based phototransistor for room-temperature long-wavelength detection. *Sci. Adv.* **6**, eabb6500 (2020).

8 Yan, J. W. *et al.* Room-temperature THz detection via EIW effect based on graphite nanosheet. *J. Infrared Millim. W.* **41**, 551-556 (2022).

9 Ma, W. L. *et al.* Detection of Long Wavelength Photons via Quasi-Two-Dimensional Ternary Ta_2_NiSe_5_. *ACS Appl. Electron. Ma.* **4**, 2979-2986 (2022).

10 Dong, Z. *et al.* Excitonic Insulator Enabled Ultrasensitive Terahertz Photodetection with Efficient Low-Energy Photon Harvesting. *Adv. Sci.* **9**, 2204580 (2022).

11 <https://www.tydexoptics.com/pdf/Golay_Detectors.pdf>

12 https://electronics.leonardo.com/documents/16277707/18362607/DLATGS_IR_Detectors_for_Instrumentation_LQ_mm08096_b.pdf?t=1538987888860

13 https://www.lasercomponents.com/fileadmin/user_upload/home/Datasheets/lc-pyros/d31-lt31-vm-pyro-detectors.pdf

14 Guo, T. T. *et al.* High-Gain MoS_2_/Ta_2_NiSe_5_ Heterojunction Photodetectors with Charge Transfer and Suppressing Dark Current. *Acs Appl. Mater. Inter.* **14**, 56384-56394 (2022).

15 Zhang, Y. *et al.* Two-dimensional Ta_2_NiSe_5_/GaSe van der Waals heterojunction for ultrasensitive visible and near-infrared dual-band photodetector. *Appl. Phys. Lett.* **120**, 261101 (2022).

16 Xiao, P. *et al.* Visible Near-Infrared Photodetection Based on Ta_2_NiSe_5_/WSe_2_ van der Waals Heterostructures. *Sensors* **23**, 4385 (2023).

17 Chen, J. P. *et al.* High-performance self-powered ultraviolet to near-infrared photodetector based on WS_2_/InSe van der Waals heterostructure. *Nano Res.* **16**, 7851–7857 (2023)

18 Huang, Z. H. *et al.* Integration of photovoltaic and photogating effects in a WSe_2_/WS_2_/p-Si dual junction photodetector featuring high-sensitivity and fast-response. *Nanoscale Advances* **5**, 675-684 (2023).

19 Fang, C. C. *et al.* WS_2_/Bi_2_O_2_Se van der Waals Heterostructure with Straddling Band Configuration for High Performances and Broadband Photodetector. *Adv. Mater. Interfaces* **9**, 2102091 (2022).

20 Qi, Z. D. *et al.* Epitaxy of NiTe_2_ on WS_2_ for the p-Type Schottky Contact and Increased Photoresponse. *ACS Appl. Mater. Inter.* **14**, 31121-31130 (2022).

21 Wu, D. *et al.* Ultrabroadband and High-Detectivity Photodetector Based on WS_2_/Ge Heterojunction through Defect Engineering and Interface Passivation. *Acs Nano* **15**, 10119-10129 (2021).

22 Zhang, S. *et al.* H-BN-Encapsulated Uncooled Infrared Photodetectors Based on Tantalum Nickel Selenide. *Adv. Funct. Mater.* **33**, 2305380 (2023).

23 Guo, T. T. *et al.* High-performance flexible broadband photodetectors enabled by 2D Ta_2_NiSe_5_ nanosheets. *2D Mater.* **10**, 025004 (2023).

24 https://www.hamamatsu.com.cn/content/dam/hamamatsu-photonics/sites/documents/99_SALES_LIBRARY/ssd/s16586_kspd1094e.pdf

25 https://www.hamamatsu.com.cn/content/dam/hamamatsu-photonics/sites/documents/99_SALES_LIBRARY/ssd/g6854-01_kird1013e.pdf

26 Huang, Z. M. *et al.* Extreme Sensitivity of Room-Temperature Photoelectric Effect for Terahertz Detection. *Adv. Mater.* **28**, 112-117 (2016).

27 Huang, Z. M. *et al.* Room-Temperature Photoconductivity Far Below the Semiconductor Bandgap. *Adv. Mater.* **26**, 6594-6598 (2014).

28 Rogalski, A. *Infrared detectors*. (CRC Press, 2011).

29 Tang, T. W. *et al.* Non-Coulomb strong electron-hole binding in Ta_2_NiSe_5_ revealed by time- and angle-resolved photoemission spectroscopy. *Phys. Rev. B* **101**, 235148 (2020).

30 Xiao, W. Z. *et al.* Two-dimensional H-TiO_2_/MoS_2_ (WS_2_) van der Waals heterostructures for visible-light photocatalysis and energy conversion. *Appl. Surf. Sci.* **504**, 144425 (2020).
